# Graphene (GNP) reinforced 3D printing nanocomposites: An advanced structural perspective

**DOI:** 10.1016/j.heliyon.2024.e28771

**Published:** 2024-03-27

**Authors:** AKM Asif Iqbal, Clement Stefano Harcen, Mainul Haque

**Affiliations:** a Department of Mechanical, Materials and Manufacturing Engineering, Faculty of Science and Engineering, University of Nottingham Ningbo China, 199, Taikang East Road, Yinzhou, Ningbo, 315100, China; b Department of Mathematical Sciences, University of Nottingham Ningbo China, 199 Taikang East Road, Yinzhou, Ningbo 315100, China

**Keywords:** Nanocomposite, 3D printing, Graphene, Mechanical properties

## Abstract

The influence of macro-micro structural design on the mechanical response of structural nanocomposites is substantial. The advancement of additive manufacturing especially three-dimensional (3-D) printing technology offers a promising avenue for the efficient and precise fabrication of multi-functional low-weight and high-strength nanocomposites. In contemporary discourse, there is a notable emphasis on carbon-based nanomaterials as nanofillers for structural composites due to their substantial specific surface area and exceptional load-bearing ability in mechanical structures. Notably, graphene, a distinctive two-dimensional (2-D) nanomaterial, exhibits very large elastic modulus and ultimate strength as well as remarkable plasticity. The utilization of graphene nanoparticles (GNPs) in the field of 3-D printing enables the production of intricate three-dimensional structures of varying sizes and configurations. This is achieved through the macro-assembly process, which facilitates the creation of a well-organized distribution of graphene and the establishment of a comprehensive physical network through precise micro-regulation. This paper presents an overview of multiscale structural composites that are strengthened by the incorporation of graphene and prepared by 3-D printing. The composites discussed in this study encompass graphene-polymer composites, graphene-ceramic composites, and graphene-metal composites. Furthermore, an analysis of the present obstacles and potential future advancements in this rapidly expanding domain is anticipated.

## 1 Introduction

The enhancement of the structure and performance demands of prominent components in severe environment applications has led to the recognition of lightweight and integration as crucial factors for successful and real-world structural nanocomposites [[1], [2], [3], [4], [5]]. Additive manufacturing commonly known as 3-D printing, is a progressive manufacturing technique utilized to produce diverse structures and intricate geometries based on three-dimensional (3-D) model data. This process was pioneered by Charles Hull in 1986 using a technology referred to as stereolithography (SLA). This breakthrough was subsequently tracked by advancements in the field, including powder bed fusion, fused deposition modeling (FDM), inkjet printing, and contour crafting (CC) [6]. The 3-D printing process operates by sequentially stacking layers using points, lines, and planes as fundamental structural elements. These layers are derived from the slicing of three-dimensional entities, resulting in continuous two-dimensional cross-sectional data [7,8]. In contrast to conventional conservative and comparable material manufacturing processes, 3-D printing offers the ability to overcome geometric limitations in structural design and enables the production of complex structural components. This technology provides several advantages, including cost reduction through on-demand layering and significant improvements in lightweight design achieved through integrated manufacturing [9,10]. Furthermore, the objective of 3-D printing is to enhance the efficiency of the printed structure in terms of its volume, precision, speed, structural homogeneity, and ability to include multiple materials. As a result, the use of 3-D printing is prevalent in several domains such as consumer electronics, intricate structural components, composite components with multiple materials, and challenging-to-manufacture parts [[11], [12], [13], [14], [15], [16]]. In particular, the consistent application of the shear effect in micro-regulation enables the achievement of an organized distribution and highly aligned arrangement of components. This process further facilitates the establishment of a mechanically reinforced network that exhibits better penetration capabilities [17,18]. Hence, the utilization of 3-D printing technology offers expeditious and cost-effective means of digitally-driven manufacturing for the creation of lightweight and robust composite structures. This has considerable practical value in terms of enhancing the strengthening mechanism of structural nanocomposites. The design of component configuration is a fundamental determinant in attaining significant structural performance [19]. At now, the use of printed components in structural composites mostly involves the incorporation of thermoplastic polymers with metallic and ceramic powders. The resultant component often has exceptional mechanical stability and a discernible toughening effect. However, the porous structure created by 3-D printing reveals a notable reduction in strength, posing challenges in meeting the demands for lightweight and greater strength-oriented applications. Hence, carbon-based nanomaterials have garnered significant attention as nanofillers for structural composites due to their substantial specific surface area and exceptional mechanical characteristics [[20], [21], [22]]. This is particularly true for graphene, a two-dimensional (2-D) substance composed of carbon atoms organized in a single layer and in a honeycomb lattice orientation [[23], [24], [25], [26], [27]]. The heightened mechanical qualities of 3-D-printed graphene-reinforced nanocomposites can be attributed to the strengthening processes shown by graphene nanoparticles (GNPs), such as fracture deflection, bridging, and nanoplatelet pull-out [[28], [29], [30]]. These features contribute to the achievement of optimum structural performance in these composites. In addition, the utilization of 3-D printing technology enables the controlled fabrication of 3-D graphene structures, which exhibit a robust network for enhancing toughness, distinctive porosity, and flexibility [31,32].

Based on the work on 3-D printing, the goal of this paper is to combine the up-to-date outcomes in the application of 3-D-printed composites strengthened with graphene for structural applications. Furthermore, this paper accumulates the results that show different optimization processes employed by graphene inside a range of nanocomposite materials, including polymer matrix composites (PMCs), ceramic matrix composites (CMCs), metal matrix composites (MMCs), and other structural nanocomposites often utilized in the realm of biological engineering. The integration of graphene and its derivatives, namely graphene oxide (GO) and reduced graphene oxide (rGO), with 3-D printing technology, has garnered significant interest due to the examination of their solubility in aqueous environments and the rheological characteristics of the inks used for printing. The exceptional mechanical properties of GNPs may be preserved to a significant degree when incorporating them into a three-dimensional graphene structure, resulting in unique characteristics like porosity, flexibility, and an increased specific surface area [33,34]. The GNPs serve as nano-reinforcements by securely anchoring and attaching themselves to the matrix particles. This facilitates the uninterrupted passage of stress during the deformation process. Furthermore, the utilization of 3-D printing enables the creation of a distinct multi-dimensional support structure that effectively exhibits the evident mechanical interlocking properties of the nanocomposites. Additionally, it has been shown that the oriented GNP network structure exhibits distinctive anisotropy and cohesive toughness [35]. The utilization of 3D-printed GNP-reinforced nanocomposites in the domain of structural materials demonstrates a favorable impact on the pragmatic potential of carbon-based nanomaterials as nano-strengthening elements.

## 2 3D printing process

**Fig. 1:**
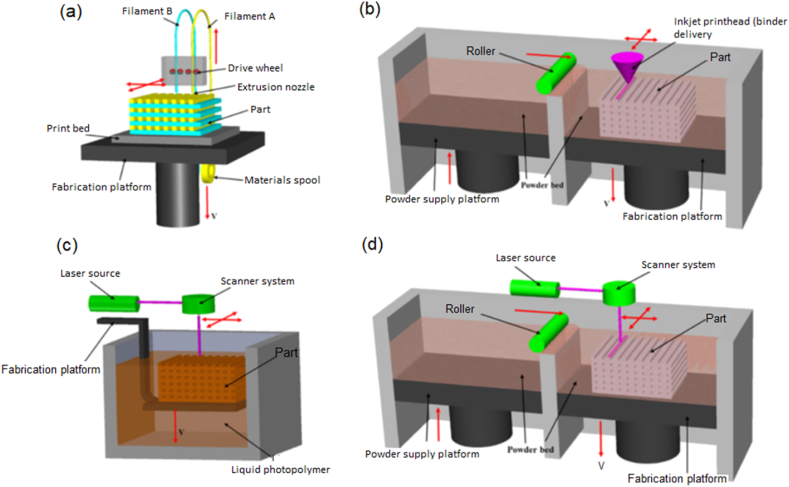
Schematic diagram of a typical (a) fused deposition modeling, FDM; (b) inkjet printing; (c) stereolithography SLA; (d) powder bed fusion [36] (reprinted with permission from Elsevier) (Reprinted with permission from Elsevier).

Numerous techniques of additive manufacturing (AM) have been devised in order to accomplish the requirement of fabricating complex structures with high precision. The advancement of additive manufacturing (AM) technologies has been propelled by numerous significant variables, including rapid prototyping, the volume to print sizable structures, the mitigation of production defects, and the upgrading of mechanical features. Fig. 1 presents schematic illustrations illustrating the four primary techniques employed in additive manufacturing: (a) fused deposition modeling, (b) inkjet printing, (c) stereolithography, and (d) powder bed fusion [36]. Fused deposition modeling (FDM) is the prevailing technique in 3D printing, mostly employing polymer filaments. Furthermore, the primary methods of additive manufacturing (AM) include selective laser sintering (SLS), selective laser melting (SLM), liquid binding in three-dimensional printing (3DP), inkjet printing, contour crafting, stereolithography, direct energy deposition (DED), and laminated object manufacturing (LOM). The subsequent paragraphs provide concise explanations of these approaches, along with an introduction to their applications and appropriate materials. Additionally, the advantages and disadvantages of each method are examined.

### 2.1 Fused deposition modeling (FDM)

The Fused Deposition Modeling (FDM) approach involves the utilization of an incessant filament composed of a thermoplastic polymer to fabricate successive coatings of materials, as shown in Fig. 1a. The filament undergoes heating at the nozzle in order to attain a partially liquefied condition, following which it is extruded onto the platform or onto pre-existing layers. The thermoplastic nature of the polymer filament is a crucial characteristic in this process, as it enables the filaments to undergo fusion during the printing phase and subsequently harden at ambient temperature post-printing. The mechanical characteristics of printed items are primarily influenced by the layer thickness, breadth, and alignment of filaments, as well as the presence of air gaps within the same layer or across layers [37]. The primary factor contributing to mechanical weakness was determined to be inter-layer alteration [38]. The main benefits of FDM include its affordability, rapid production capabilities, and user-friendly nature. However, FDM has several limitations in terms of its mechanical qualities, layer-by-layer structure, surface quality [39], and a restricted range of thermoplastic materials [37]. The utilization of FDM in the production of fiber-reinforced composites has resulted in enhanced mechanical characteristics of 3-D printed components [40]. Nevertheless, the primary obstacles encountered in 3-D printed composite parts are fiber coordination, fiber-matrix bonding, and void generation [40,41].

### 2.2 Inkjet printing and contour crafting

In the realm of additive manufacturing for ceramics, inkjet printing stands as a prominent technique. The technology is employed for the fabrication of intricate and sophisticated ceramic structures, primarily utilized in the field of tissue engineering for the production of scaffolds. The present technique involves the utilization of a consistent ceramic suspension, such as zirconium oxide powder dispersed in water [42]. This suspension is propelled and afterward dispensed onto the substrate in the form of droplets by an injection nozzle. The droplets subsequently undergo a process of consolidation, resulting in the formation of a cohesive pattern that attains a level of solidity capable of supporting succeeding layers of printed materials (Fig. 1b). The aforementioned approach exhibits rapidity and effectiveness, hence introducing a heightened degree of adaptability in the creation and production of intricate configurations. There are two primary categories of ceramic inks, namely wax-based inks and liquid suspensions. The process of solidifying wax-based inks involves melting them and subsequently depositing them onto a cool substrate. Conversely, the solidification of liquid suspensions occurs through the process of liquid evaporation. The quality of inkjet-printed components is influenced by several aspects, including the particle size distribution of ceramics, the viscosity of the ink, the solid content, the extrusion rate, the nozzle size, and the printing speed [43]. The primary limitations of this approach include the need to ensure workability, the presence of coarse resolution, and the absence of adhesion between layers. Contour crafting, a process akin to inkjet printing, serves as the primary modality for the additive production of expansive architectural edifices. This technique demonstrates the ability to extrude concrete paste or dirt through the utilization of bigger nozzles and elevated pressure levels. The application of contour crafting technology for lunar building has been demonstrated through prototyping [44].

### 2.3 Stereolithography (SLA)

The Stereolithography (SLA) technique, which emerged in 1986 [45], is considered one of the pioneering technologies in the field of additive manufacturing. UV light or an electron beam is employed to induce a chain reaction on a layer of resin or monomer solution. The monomers, predominantly composed of acrylic or epoxy-based materials, exhibit UV reactivity and undergo rapid polymerization upon activation by radicalization. Following the process of polymerization, the resin layer undergoes solidification, resulting in the formation of a pattern that serves the purpose of supporting the successive layers (Fig. 1c). The unreacted resin is extracted subsequent to the conclusion of the printing process. In order to attain the necessary mechanical features, certain printed components may need a post-process treatment, such as heating or photo-curing. The utilization of a monomer-based dispersion including ceramic particles has been demonstrated in the fabrication of ceramic-polymer composites [43]. Additionally, this approach has also been employed in the synthesis of polymer-derived ceramifiable monomers, such as silicon oxycarbide [46]. The SLA technology is capable of producing components with exceptional quality, characterized by a precise resolution that may reach as low as 10 μm [41]. Conversely, the printing process has a comparatively sluggish pace, entails substantial costs, and is characterized by a restricted assortment of printable materials. Moreover, the dynamics associated with the reaction and the subsequent curing process exhibit a high degree of complexity. The thickness of each layer is mostly evaluated by the energy of the light source and the intensity, as indicated by previous research [45]. The utilization of SLA has proven to be highly efficient in the fabrication of intricate nanocomposites [47].

### 2.4 Powder bed fusion

Powder bed fusion procedures include the deposition of thin layers of very fine powders, which are uniformly distributed and densely packed onto a platform. The integration of the powders inside each layer is achieved by the utilization of either a laser beam or a binder. The process involves the application of successive layers of powders, which are then rolled onto the preceding layers and fused together, ultimately resulting in the construction of the final three-dimensional component (Fig. 1d). Subsequently, the surplus powder is eliminated by the utilization of a vacuum, and other procedures such as coating, sintering, or infiltration may be conducted if deemed required. The success of this process is primarily influenced by the powder size distribution and packing since these elements play a critical role in determining the density of the printed object [48]. The utilization of lasers is limited to powders with a low melting/sintering temperature, necessitating the usage of a liquid binder for other types of powders. Selective laser sintering (SLS) exhibits versatility in accommodating a diverse range of polymers, metals, and alloy powders. In contrast, SLM is limited to specific metals, namely steel and aluminum. The laser scanning technique employed in selective laser sintering (SLS) does not achieve complete powder melting. Instead, it generates localized high temperatures on the surface of the powder grains, leading to molecular fusion of the powders. In contrast, the powders undergo complete melting and fusion upon laser scanning in selective laser melting (SLM), leading to the attainment of enhanced mechanical characteristics [49]. A comprehensive examination of various materials and applications utilizing SLM may be accessed in the reference provided by Yap et al. [50].

When a liquid binder is employed, the process is commonly known as three-dimensional printing (3D printing). The chemical composition and flow properties of the binder, as well as the size and morphology of the powder particles, the rate at which deposition occurs, the interplay between the powder and binder, and the methods employed during post-processing, all have significant implications in the field of 3D printing [41,48]. The porosity of components produced using binder deposition is often greater in comparison to laser sintering or melting techniques, which have the capability to fabricate solid pieces [48]. The primary factors that influence the sintering process are the laser power and scanning speed. Further details on different types of lasers and their impact on 3D printing may be found in the article of Lee et al. [49]. The primary benefits of powder bed fusion are its ability to achieve fine resolution and high-quality printing, rendering it well-suited for the fabrication of intricate structures. The utilization of this technique is prevalent throughout diverse sectors, including sophisticated applications such as tissue engineering scaffolds, lattices, aircraft, and electronics. One notable benefit of employing this technique is the utilization of the powder bed as a means of support, therefore circumventing challenges associated with the removal of supporting material. Nevertheless, powder bed fusion, despite its sluggish nature, is accompanied by significant disadvantages such as exorbitant expenses and increased porosity resulting from the fusing of powder with a binder.

### 2.5 Direct energy deposition (DED)

The utilization of direct energy deposition (DED) has been employed in the production of high-performance super-alloys [51]. This technique is sometimes referred to as laser-engineered net shaping (LENS, laser solid forming (LSF), directed light fabrication (DLF), direct metal deposition (DMD), electron beam additive manufacturing (EBAM), and wire + Arc additive manufacturing (WAAM). The Direct Energy Deposition (DED) process employs a focused energy source, such as a laser or electron beam, to target a specific area on the substrate. This energy source is also utilized to melt the feedstock material, which can be in the form of either powder or wire, concurrently. The molten substance is thereafter placed and integrated into the molten base material and solidifies following the motion of the laser beam [51]. One notable distinction between the DED and SLM procedures is the absence of a powder bed in DED. In DED, the feedstock material is melted prior to deposition, following a layer-by-layer approach akin to Fused Deposition Modeling (FDM). However, it is important to note that DED employs far greater energy levels to achieve the melting of metal materials. Hence, the utilization of this technique might be advantageous in addressing fissures and enhancing the structural integrity of fabricated components that are constrained by the limitations of the powder-bed approach. The technique described enables the simultaneous deposition of several materials and the use of numerous axes [52]. Furthermore, the utilization of DED may be seamlessly integrated with traditional subtractive manufacturing techniques in order to achieve comprehensive machining capabilities. The aforementioned technology is frequently employed in aerospace applications, namely with materials like titanium, Inconel, stainless steel, aluminum, and their respective alloys. Generally, DED is distinguished by its notable velocities, ranging from 0.5 kg/h for Laser Engineered Net Shaping (LENS) [52] to 10 kg/h for Wire Arc Additive Manufacturing (WAAM) [53]. Additionally, DED exhibits substantial work envelopes, reaching dimensions as big as 6 m × 1.4 m x 1.4 m for printers used in commercial applications [52]. Nevertheless, it is worth noting that this particular method exhibits a reduced level of precision, with an accuracy of 0.25 mm. Additionally, it produces surfaces of inferior quality and is limited in its ability to fabricate intricate components when compared to the more advanced techniques of Selective Laser Sintering (SLS) or Selective Laser Melting (SLM) [51] Hence, DED is frequently employed in the fabrication of sizable components characterized by less intricacy, as well as in the restoration of bigger components. Dissimilar electrodeposition (DED) has the potential to effectively decrease both production time and cost, while also offering advantageous mechanical qualities, precise control over microstructure, and perfect management of composition. The aforementioned technique is applicable for the restoration of turbine engines and other specialized uses within diverse sectors, including the automobile and aerospace industries.

### 2.6 Laminated object manufacturing (LOM)

Laminated object manufacture (LOM) is considered to be among the initial additive manufacturing techniques that were made accessible for commercial use. This technology operates by systematically cutting and layering sheets or rolls of materials. The process involves the accurate cutting of consecutive layers using either a mechanical cutter or a laser, followed by their subsequent bonding in a form-then-bond manner or vice versa, in a bond-then-form manner. The form-then-bond process is particularly helpful for the thermal bonding of ceramics and metallic materials, which also enables the building of interior features by eliminating superfluous materials before bonding. The surplus materials remaining after the cutting operation are retained for support purposes and can subsequently be eliminated and recycled [54]. The LOM technique has demonstrated its applicability across a range of materials, including polymer composites, ceramics, paper, and metal-filled tapes. Post-processing such as high-temperature processing may be necessary based on the materials type and desired performance. Ultrasonic additive manufacturing (UAM) is a recently developed category within the realm of laminated object manufacture (LOM). It involves the integration of ultrasonic metal seam welding and computer numerical control (CNC) machining techniques during the lamination process [55]. The unique capability of UAM lies in its ability to produce metal structures at low temperatures, making it the sole additive manufacturing process with this characteristic [56,57]. The utilization of LOM has been observed across many sectors, including paper manufacturing, foundry industries, electronics, and smart buildings. Smart structures are categorized as structures that include several functionalities, owing to their incorporation of numerous sensors and processors. In contrast to traditional approaches, Urban Air Mobility (UAM) has the capability to identify and include void spaces within a building through the utilization of integrated computer-aided design. This enables the accommodation of embedded electrical devices, sensors, pipelines, and other relevant components. Electronic devices have the capability to be fabricated using the lamination process of Ultrasonic Additive Manufacturing (UAM) by employing direct write technologies [54]. Laser-based powder bed fusion (LBM) is capable of yielding significant reductions in tooling expenses and manufacturing duration, rendering it a highly effective additive manufacturing technique particularly suited for the production of large-scale structures. Nevertheless, it should be noted that Laser on Melting (LOM) has substandard surface quality in its raw state and exhibits poorer levels of dimensional precision when compared to powder-bed techniques. Moreover, the process of eliminating surplus components of laminates subsequent to the object's production is more time-intensive in comparison to powder-bed techniques. Hence, it is not advisable for intricate geometrical configurations. Table 1 presents a comprehensive overview of the principal techniques employed in additive manufacturing.

**Table 1:** Key elements of major 3D printing techniques.

Methods	Materials Use	Applications	Advantages	Limitations	Resolution range (μm)	Ref:
Fused deposition modeling (FDM)	Continues filaments of thermoplastic polymers, Continuous fiber-reinforced polymers	Advanced composite parts, rapid prototyping, toys,	High-speed, Simple, low cost	Only thermoplastic can be used, weak mechanical properties	50–200 μm	[41]
Inkjet printing and contour crafting	Concentrated distribution of particles in a liquid (ink or paste) Ceramic, concrete, and soil	Biomedical, large structures, buildings	Big structures can be printed, Fast printing	Lack of adhesion between layers, coarse resolution, maintaining workability,	Inkjet: 5–200 μm, Contour crafting: 25–40 mm	[58]
Stereolithography (SLA)	Resin, Hybrid polymer-ceramics	Prototyping biomedical,	High quality high-resolution,	Very limited materials, slow printing, expensive	10 μm	[41]
Powder bed fusion (SLS, SLM, 3DP)	Fine metal powder, alloy powder, polymers (SLS or SLM), ceramic and polymers (3DP)	Light structures, electronics, biomedical, heat exchangers	High-resolution, high-quality	Slow printing, expensive, high porosity in the binder method (3DP)	80–250 μm	[41]
Direct Energy Deposition (DED)	Powdered Metals and alloys, Ceramic and polymer wire	Aerospace, retrofitting, repair cladding, biomedical	Composition and microstructure can be controlled, Exceptional for repair and retrofitting, need less time, cheap,	Require a dense support structure, low accuracy, low surface quality	250 μm	[51]
Laminated Object Manufacturing (LOM)	Polymer composites, Ceramics, Paper Metal-filled tapes, Metal rolls	Foundry industries, electronics, paper manufacturing, smart structures	A vast range of materials can be used, less tooling and manufacturing time, low cost, excellent for manufacturing large structures	Difficulty in manufacturing complex shapes, Inferior surface quality, and dimensional accuracy.	Depends on the thickness of the laminates	[51]

## 3 3-D printed structural composites

Nowadays, Composite materials are highly desirable for structural applications due to their unique combination of properties that offer superior performance compared to traditional materials. Composites are typically composed of two or more distinct materials such as fibers embedded in a matrix, working synergistically to create a material with enhanced strength, stiffness, and durability. One key advantage is the exceptional strength-to-weight ratio that composites provide, making them significantly lighter than many traditional materials while maintaining comparable or even superior strength [[59], [60], [61]]. This characteristic is particularly advantageous in industries such as aerospace and automotive, where reducing weight is critical for fuel efficiency and overall performance. Additionally, composites exhibit excellent corrosion resistance, a crucial attribute in applications exposed to harsh environmental conditions. Their versatility in design and ability to be tailored to specific performance requirements make composites an ideal choice for structural components, offering a compelling solution to the evolving demands of modern engineering across various sectors [62,63]. The advent of 3D-printed structural composites is a pivotal development that addresses longstanding challenges in material design and manufacturing. By incorporating advanced materials like carbon fiber or graphene in the 3D printing process, it is possible to precisely control the arrangement of fibers within the structure, optimizing strength and durability. This level of customization allows for the creation of components with tailored mechanical properties, making them ideal for applications where specific performance requirements are crucial [40]. Furthermore, the efficiency of the 3D printing process reduces material waste, contributing to sustainability goals in various industries. As research and development in this field continue to progress, the potential for 3D-printed structural composites to revolutionize traditional manufacturing processes becomes increasingly evident. The adaptability, efficiency, and performance benefits offered by this technology underscore its transformative impact on the design and production of structural components in diverse sectors. In the following paragraphs, we will discuss three major categories of 3-D printed composite materials that have been prepared for structural applications.

### 3.1 3D printed graphene-polymer composites

The exceptional mechanical characteristics shown by GNPs, including their elevated Young's modulus, exceptional breaking strength, and notable stretchability, render them highly suitable as nanofillers in the fabrication of polymer matrix composites with enhanced strength [[64], [65], [66]]. The incorporation of GNPs into polymer monomers followed by polymerization results in the formation of a nanocomposite system. In this system, the close arrangement of GNPs inside the polymer matrix has a beneficial impact on enhancing the strength and plasticity of the nanocomposites. The incorporation of GNP into polymer bases offers a wide range of functions, enhancing their processing performance and expanding their potential applications in structural contexts [[67], [68], [69], [70]]. The incorporation of GNPs into polymer composites has resulted in notable enhancements in mechanical, thermal, electrical, and flame-retardant properties, as demonstrated by comparative studies with pure polymer monomers [71]. Bora et al. [72] introduced a nanocomposite consisting of a polyester resin matrix reinforced with GNP, resulting in enhanced ultimate strength and elastic modulus. This improvement may be due to the effective dispersion of GNPs within the matrix and their interaction with the surrounding material. The research team led by Valiyaveettil successfully produced ultrathin nanocomposite films consisting of GNP and polyvinyl chloride (PVC). These films demonstrated a significant enhancement in elastic modulus, with a 58% increase, as well as a notable gain in tensile strength, with a 130% enhancement. Remarkably, these improvements were achieved by including just 2 wt% loadings of graphene into the composite films [73]. It is important to acknowledge that even a minimal quantity of GNP loading can yield significant enhancements in the mechanical performance of the nanocomposite structure.

In order to effectively utilize GNP-strengthened polymer matrix composites in various structural applications, it is crucial to employ appropriate multi-dimensional structural design and carefully choose components. This is necessary to achieve optimal performance, as highlighted in previous studies [14,74]. The high viscosity and modulus shown by some graphene and polymer monomers make them well-suited for 3-D printing. Additionally, the free design features of these materials can further emphasize the structural performance benefits of nanocomposites [15,[75], [76], [77]]. In contrast to the composite structure formed through the process of polymer encapsulation of porous 3-D GNP, the utilization of mixed or eutectic GNP/polymer inks in 3-D printing enables the achievement of homogeneous dispersion and direct interaction between GNP and the polymer matrix. This approach enhances the beneficial impact of GNP on the structural performance of nanocomposites [[78], [79], [80]].

**Fig. 2:**
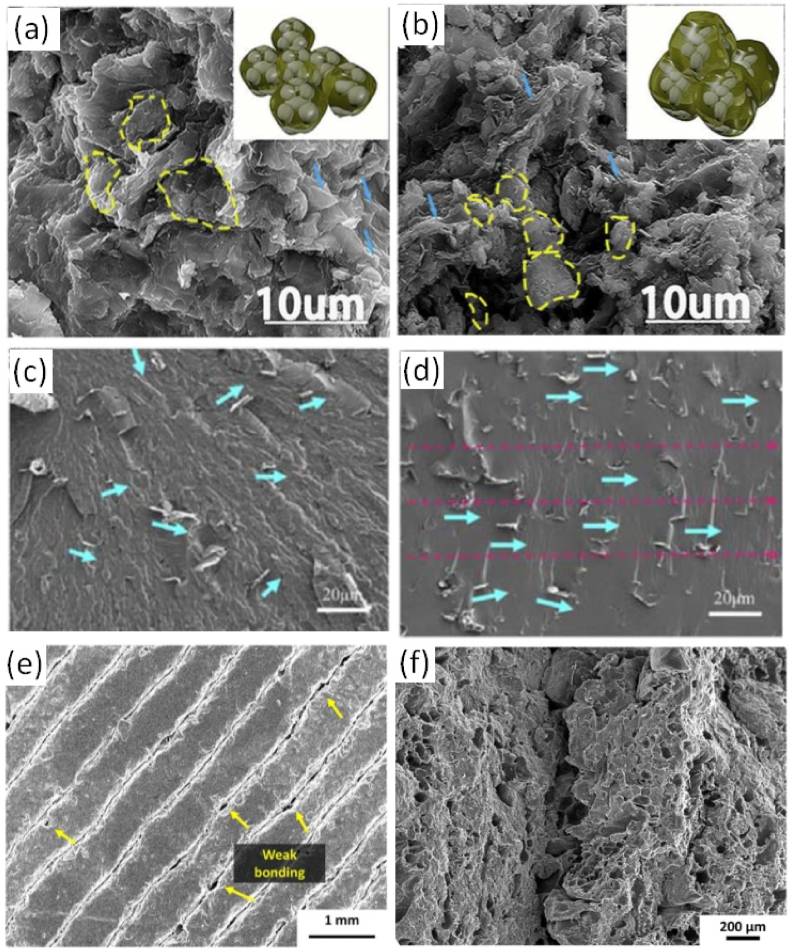
SEM image of (a) (b) GO/Geopolymer nanocomposite showing the reduction of particle agglomeration with the increase of GO content in the GO/Geopolymer composite [81] (c) (d) cross-section of 3D printed 2% graphene-polymer composite showing the (c) random orientation of graphene (d) ordered orientation of graphene [82] (Reprinted with permission from Elsevier) (e) microstructure of 3D printed PLA-GNP composite showing a higher layer thickness (f) Fractured cross-sectional microstructure of 3D printed PLA-graphene [83] (Reprinted with permission from Willey).

**Fig. 3:**
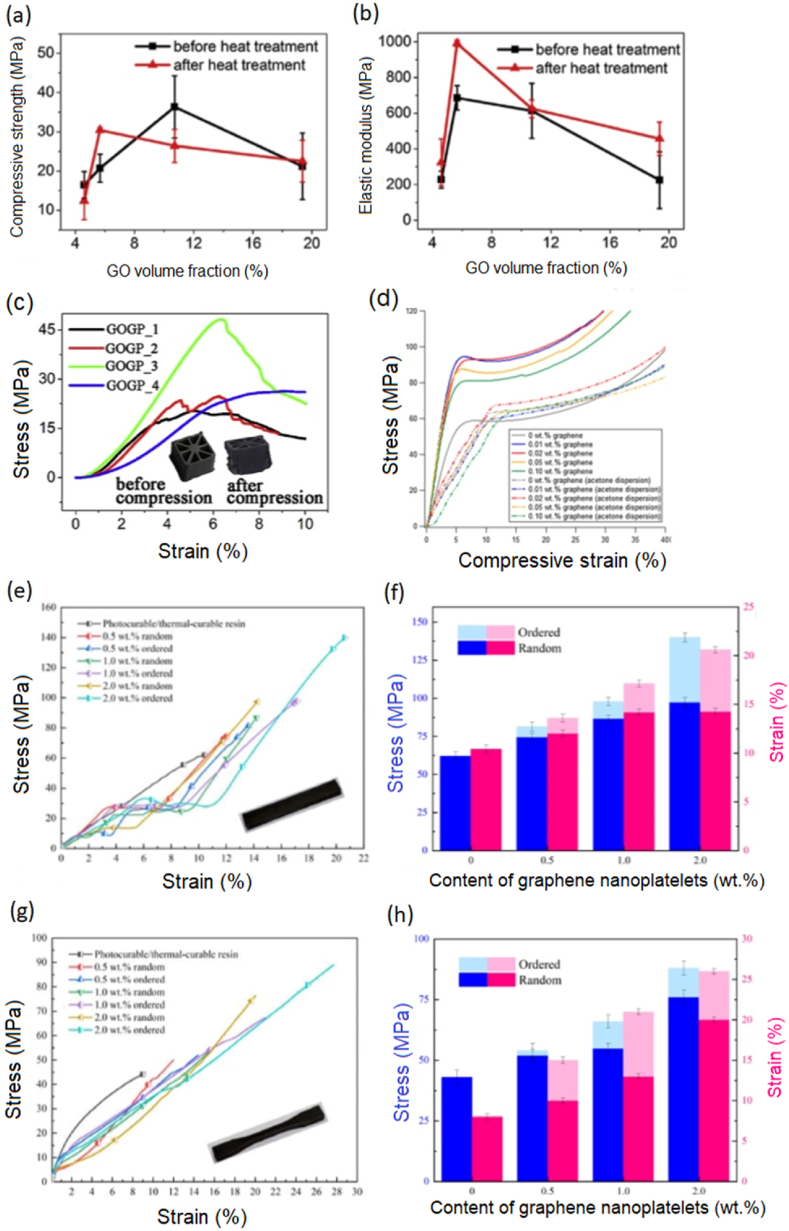
(a–c) Mechanical properties including compressive strength, elastic modulus and stress-strain pattern of GO/Geopolymer (GOGP) nanocomposite, all the mechanical strength decreases at higher volume fraction of GO as higher volume fraction creates agglomeration of GO in the GOGP composite [81], (d) Stress-strain curve of 3D printed (using the stereolithographic, SLA technique) graphene-polymer composite with or without dispersion of acetone showing the reduction of Young's modulus and Yield strength of the acetone dispersed composites [75]. (e–h) mechanical characteristics of random and ordered composites containing varying weight percent of graphene ranging from 0 to 2 wt%: **(e)** the bending stress-strain curves (f) the flexural strength and fracture strain (g) the tensile stress-strain curves and (h) tensile strength and elongation [84] (Reprinted with permission from Elsevier).

Fig. 2 demonstrates the SEM micrograph of 3D printed GNP-polymer composites showing the uniformity of the dispersion of graphene in the polymer matrix and Fig. 3 illustrates the mechanical properties of different 3-D printed GNP-polymer composites. Architectural designability plays a crucial role in enhancing the potential utilization of composites inside essential and intricate structural elements. The scaffold structure with a graded porosity was fabricated by Bustillos and his research group using a composite material composed of 3-D-printed GNP-reinforced polylactic acid (GNP-PLA nanocomposite) [83]. The uppermost layer of the printed nanocomposite scaffolds exhibited a greater thickness of about 0.48 ± 0.05 mm (as shown in Fig. 2e). compared to that of 3-D-printed monolithic PLA. This disparity may be ascribed to a combination of the thermal conduction properties of graphene and the specific processing conditions employed. The increased heat conductivity shown by graphene gives rise to a thermal strain mismatch, which subsequently hampers the effectiveness of intralayer and interlayer bonding in GNP-PLA scaffolds. A study showed that GNP-PLA composites have reduced creep displacement during primary creep in comparison to single PLA [85]. The observed enhancement in creep resistance in Bastillos's study can be attributed to the obstructive influence exerted by the graphene nanofiller on the process of strain hardening. This study has shown that the incorporation of GNPs into the composites resulted in a notable reduction of around 20.5% in creep displacement. This reduction may be attributed to the synergistic effects of strain hardening and recovery processes [83]. Additionally, an assessment was conducted on the wear characteristics of composites made from 3-D printed GNP-PLA materials. It is observed that during the initial 10-min period, the GNP-PLA nanocomposites exhibited a comparatively lower coefficient of friction (COF) ranging from 0.1 to 0.35. Subsequently, the COF gradually increased to 0.55, which was comparable to those observed in pure PLA, following a transitional phase. Furthermore, the decreased average width of the wear track seen in GNP-PLA composites serves as an indication of their enhanced wear resistance. The viability of utilizing 3-D-printed GNP-PLA nanocomposites in structural applications is demonstrated through the optimization of creep and wear resistance. The utilization of 3D printing technology for regulating the particular architecture of GNP-reinforced polymer nanocomposites is well recognized as a significant practice for enhancing their structural characteristics [[86], [87], [88]]. Lai and Markandan [75] conducted a study on the GNP-polymer octet-truss lattices, wherein they observed enhanced modulus and strength. These improvements were due to the inclusion of aligned GNPs. Lee and his co-workers developed a wire with a flexible and reconfigurable structure, characterized by a "*S*" shape, using GNP-PLA composites. This wire demonstrated enhanced mechanical capabilities, with a 44% increase in tensile strength and a 57% increase in maximum strain [89]. Additionally, the interaction between GNPs and the polymer matrix plays a crucial role in enhancing the structural features of nanocomposites. The micro-evolution process of this bonding state has been investigated by Zhong et al. [81]. The authors presented the novel porosity architectures of a geopolymer/GO nanocomposite, highlighting the significant interaction between GO and hydrated geopolymer particles (HGPPs) [81]. The inclusion of graphene oxide (GO) resulted in enhanced rheological characteristics of geopolymer. The fracture plane of the nanocomposite with 4 wt% GO loading exhibited a rather smooth appearance. Additionally, it was observed that all the high glass transition temperature polymer particles (HGPPs) were fully enclosed within the GO films. By increasing the loading of graphene oxide (GO), it is possible to observe the fracture plane and edges of GO sheets. Moreover, the researchers developed free-standing structures by arranging layers in a random manner and splitting high-grade polypropylene particles (HGPPs) into smaller pieces. The relationship between the microstructures and the structural activities of the printed nanocomposites is influenced by the increasing loading of GO. The nanocomposite with 10 wt% loading of graphene oxide (GO) exhibited notable compressive strength and elastic modulus values, reaching 36 MPa and 700 MPa, respectively. These enhanced mechanical properties can be attributed to the adhesive properties of the GO layers. The graphene oxide (GO) layers possess a robust adhesive property in the two-dimensional (2-D) form, enabling them to effectively integrate with high-performance glassy polymer particles (HGPPs) and promote a strong connection between the two materials. Moreover, the observed reduction in the strength and modulus of the nanocomposite containing 20 wt% loadings of graphene oxide (GO) might be primarily attributed to the inevitable agglomeration of nanoparticles. This study investigated the utilization of graphene oxide (GO) as a modulator of rheological properties through an "encapsulation mechanism." It also presented a potential avenue for the development of novel structural composites using non-printable polymer matrix materials. The enhancement of structural performance in composites may be achieved by the connection of GNPs with a polymer matrix. In addition, the conformation of these composites can be supervised by leveraging the designability of 3-D printing, hence expanding their potential applications [[90], [91], [92]]. The use of the metastable and temperature-dependent structure of GO, alongside GNP, is employed to augment the structural capabilities of polymer-based nanocomposites. Manapat et al. [93] conducted a study in which they fabricated a resin using Stereolithography (SLA) printing technology, incorporating a small quantity of GO nanofillers. The researchers observed a significant increase in the tensile strength of the resin, reaching a value of 673.6% when annealed at a temperature of 100 °C. This enhancement may be attributed to the unique metastable structure of GO and the better interaction between the GO nanofillers and the resin matrix [93]. Despite being considered an efficient approach for the grounding of high-performance polymer nanocomposites, the presented routines encounter significant obstacles. These issues encompass the dispersibility of GNPs, the shrinking of the polymer matrix, and the optimization threshold of GNPs. Hence, it is important to consider the dispersal state of GNP inside the polymer matrix in future studies. This aspect has significance in ensuring the homogeneity of structural features and mitigating stress concentration. In addition, it is important to note that the curing process of composites can lead to significant contraction and deformation of the polymer matrix, which can compromise the structural integrity of the material. Therefore, it is crucial to provide a stable curing process to mitigate these issues. The enhancement effect of the structural performance of graphene often exhibits a threshold, whereby the extreme loading has a favorable impact. Consequently, it is imperative to exercise caution in regulating the graphene concentration within composites.

### 3.2 3-D printed graphene-ceramic composite

The performance criteria for ceramic matrix composites (CMCs) in crucial structural elements of aero-engines have become increasingly demandable due to the rapid advancements in aerospace, and military equipment industries. These criteria include high strength-to-weight ratio, high elastic modulus, and near-ductile failure [[94], [95], [96], [97]]. In conventional CMCs reinforced with fibers, the weaving of fibers at the micro-level enables the activation of strengthening and toughening mechanisms like bridging and pull-out. These mechanisms contribute to the enhancement of fracture toughness in ceramic composites, ultimately leading to a substantial optimization of their mechanical characteristics [[98], [99], [100]]. Nevertheless, the toughening mechanism in question is solely dependent on micro-scale fibers, thereby posing a challenge in achieving nano-scale toughness for ceramic particles. Consequently, the ceramic is prone to generating a significant concentration of micro-level cracks under minimal stress, thereby leading to a subsequent decline in the overall mechanical properties of this type of composite [101]. Hence, it is imperative to enhance the structural ability of the micro-domain ceramic by using a more refined strengthening and toughening process.

The incorporation of carbon-based nanoparticles into CMCs has emerged as a promising approach for enhancing the structural and functional properties of these composites across various scales [[102], [103], [104]]. As an illustration, Tapaszto's research team utilized carbon nanotubes (CNT) to enhance the mechanical strength of Si_3_N_4_ composites. This was achieved through the in-situ growth of multi-walled CNTs, resulting in composites with a flexural strength of 649 MPa in three-point bending and 548 MPa in four-point bending [105]. In a similar vein, Belmonte et al. studied a mechanical interlock mechanism for GNP-reinforced SiC composites. This mechanism led to a 162% increase in fracture toughness and a 60% increase in strength compared to monolithic SiC [106]. The utilization of GNPs as a nanofiller in composites has significant potential due to their superior properties [[107], [108], [109]]. Nevertheless, the conventional π-π interaction observed between layers of graphene serves to promote the formation of irreversible agglomeration and stacking, hence leading to the development of local stress concentration within the bulk material. The presence of nano-reinforcement in the composites, which is unevenly distributed, might result in the formation of non-uniformly sized holes. This, in turn, can cause a notable reduction in the mechanical strength of the material [110,111]. Hence, achieving uniform dispersion of GNPs inside the ceramics and establishing intimate contact between the two components are critical factors for attaining optimal strength in CMCs. The in-situ synthesis of GNPs on ceramics is regarded as a viable method for incorporating 2-D nanostructures into composites. This approach allows for precise control over the amount, distribution, and alignment of the nanosheets, while also preventing their aggregation [112]. As an illustration, Ortiz et al. conducted a study whereby they fabricated fine-grained composites of graphene and SiC using the process of spark-plasma sintering (SPS). The incorporation of epitaxially grown GNP flakes in these composites was found to greatly enhance their resistance to cone/ring cracking and improve their overall toughness [113]. However, it is important to note that GNP generated in-situ often exhibits a structure consisting of many layers. In addition, the growth sites of graphene are not permanent but rather localized on the surface layer of the materials. This poses a challenge in using the structural strengthening mechanism for CMCs. The advent of 3-D printing technology has enabled the fabrication of structural composites with customizable components through the design of raw materials. The manufacturing process facilitates the consistent distribution of GNPs throughout the composites, while also establishing a strong bond between the GNPs and the ceramic particles. In 2016, Miranzo et al. published a notable study on the utilization of 3-D printed GNP-reinforced CMCs to enhance structural performance [114]. A lightweight 3-D cellular scaffold composed of GNP-SiC was fabricated by the process of pressureless Spark Plasma Sintering (SPS). The elemental powders and sintering additives were evenly distributed by a process of repetitive stirring and sonicating. The resulting mixture was then dried and passed through a filter. Subsequently, the dispersants polyethylenimine and methylcellulose were employed to make printable inks containing evenly distributed GNPs, with the former serving as a dispersant and the latter as a viscosifying agent. To achieve the characteristic pseudoplastic behavior, it is crucial to ensure the steadiness of the printed structure and the ease of the extrusion process. The critical factors in achieving this behavior are maintaining an appropriate viscosity and inducing a shear-thinning effect. Prior to the printing process, the flat alumina substrates underwent immersion in a paraffin oil bath in order to mitigate the occurrence of drying and contraction of the printed scaffold. The inks were arranged in a stacked manner, with each layer being placed at a predetermined distance from the equivalent layer above and below it. Additionally, there was a specific spacing between adjacent filaments within each layer. The cohesion between the filaments in adjacent layers was strong, resulting in a certain level of diffusion of the filaments from each layer into the underlying layer. Subsequently, the SPS technique was employed to solidify the 3-D scaffold that had been previously created. The process of SiC grain development involves the diffusion of matter, which leads to the filling of small gaps between neighboring rods and the subsequent formation of a robust link. A notable disparity in the dispersion pattern of GNPs inside the core/shell location has been observed. The GNPs exhibited a parallel alignment with the rod shell but a random orientation with the rod core. This behavior occurs by the formation of a yielding shell and a solid core due to the radially varying shear stress [115]. The considerable influence of the porous scaffold's mechanical strength is observed through the high specific surface area and 3-D connectivity of GNPs. The stress/strain curves display distinct stress peaks, which likely correlate to the structural failure of the particular lattice. The enhancement in strain and stress at the yield point can be attributed to the in-situ coupling of GNPs with SiC grains, which is believed to be the primary mechanism responsible for toughening. Nevertheless, the specimen containing 20 vol% GNPs exhibited greater compliance, a characteristic that can be related to the percolation threshold of the nanofiller [116,117]. The compressive strengths exhibited a positive correlation with the density of the scaffolds under investigation, which aligns with the findings seen in macroporous SiC ceramics of comparable porosity [118]. Consequently, the ceramic composites supplemented with hierarchically constructed 3-D graphene exhibit notable mechanical stability, characterized by well-structured porosity and a reduced specific weight. The optimization impact of GNP-ceramic composites made using the direct mixing approach is compromised by the interfacial mismatch caused by the difference in thermal expansion coefficients between GNP and the ceramic matrix. The presence of a mismatch in the thermal expansion coefficient can give rise to interface debonding and the production of cracks at a specific temperature. This, in turn, leads to the development of internal stress and the formation of closed micropores inside the composites. The free-standing GNP scaffolds with 3D periodic systems were initially fabricated using the process of 3-D printing. This process resulted in the formation of aligned structures and a consistent dispersion of GNPs throughout the scaffolds. Subsequently, the SiC matrix has the capability to undergo in-situ growth within the nanopores by the process of chemical vapor infiltration (CVI), leading to ultimate densification. The utilization of Ethylene glycol butyl ether (EGB) as a dispersant was employed in order to facilitate the establishment of interconnections between GNPs through π-π interactions. This resulted in the production of a concentrated GNP dispersion, successfully mitigating the accumulation of its constituent mechanisms. During the first phase, the SiC matrix exhibited a preference for deposition on the graphene layers, resulting in the formation of discrete spherical particles. This led to a further separation of the graphene and the creation of a loosely wrapped SiC layer on the plane of the printed ribbon assembly. As the deposition period increased, the dispersed granular SiC underwent a progressive process of aggregation, resulting in the formation of a layered SiC structure. This layered SiC structure exhibited an interwoven and interlinked arrangement with the GNP layers. In the meanwhile, it was seen that a substantial layer of SiC coating, measuring 24.6 μm in thickness, was successfully formed. This resulted in a notable improvement in the interfacial bonding between neighboring layers at the junction. Hence, it is apparent that the incorporation of a thick SiC wrapping layer and the establishment of a tight interface between GNP and SiC layers may effectively increase the mechanical stability of GNP-SiC nanocomposites. Upon reaching a deposition time of 100 h, the compressive strength of the GNP-SiC nanocomposites exhibited a discernible decline. This decline was credited to the prevailing failure mode of the brittle SiC breaking, rather than the hardening process associated with GNPs. The study shows that the specimens with a deposition time of 50 h and a weight percentage of 50% exhibited a yield point that was 236.4% more than that of the specimens with a deposition time of 6 h. The latter specimens were able to obtain values of up to 193 MPa. The fracture mode was attributed to brittle fracture, with consideration given to the influences of pull-out and toughening of GNPs [119,120]. Hence, the proposed methodology for fabricating GNP-SiC composites entails a two-step process that facilitates the achievement of a homogeneous and closely bonded interface, hence enhancing the mechanical properties. This approach holds promise for potential applications in critical and intricate structural elements.

**Fig. 4:**
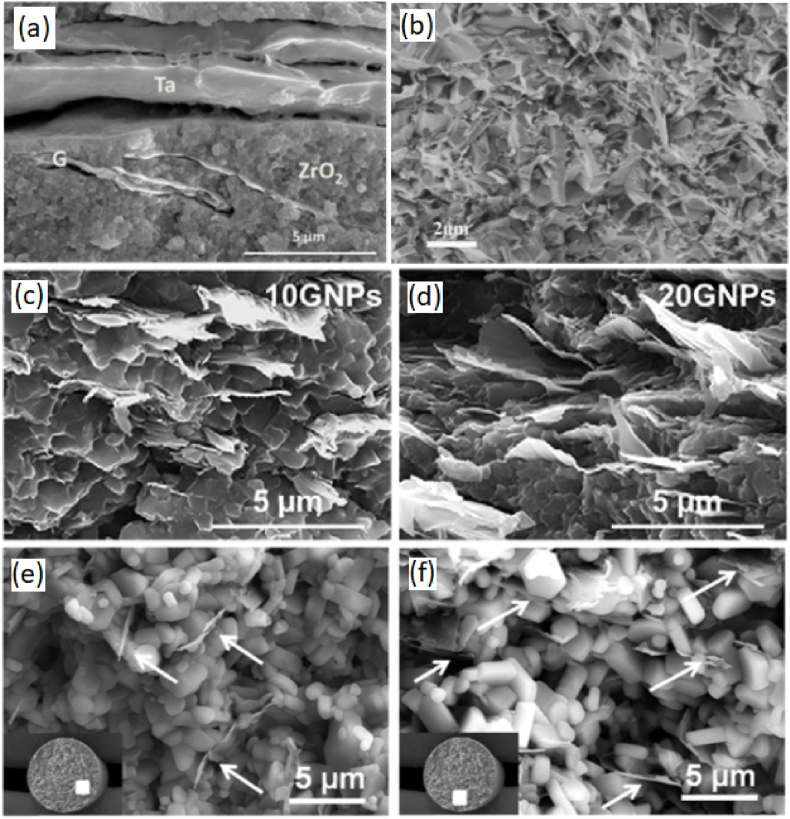
SEM image of the fracture surface of (a) SPS sintered GNP-tantalum flakes reinforced zirconia showing the bridging of the flake grains interlocking of the crack face [103]. (b) GNP- Si_3_N_4_ composite showing the lack of bulk agglomerates of GNP in the fracture surface [105]. (c) (d) FESEM micrographs of the fracture surface of SiC-GNP composites with (c) 10 vol% GNPs, and (d) 20 vol% GNPs showing the preferentially oriented GNPs in the composite [106]. (e) (f) FESEM micrographs of the fracture surfaces of GNPs-SiC cellular composites (e) 5, and (f) 10 vol% GNPs (indicated by white arrows) Showing the preferential orientation of the GNPs within the rods [114]. (Reprinted with permission from Elsevier).

**Fig. 5:**
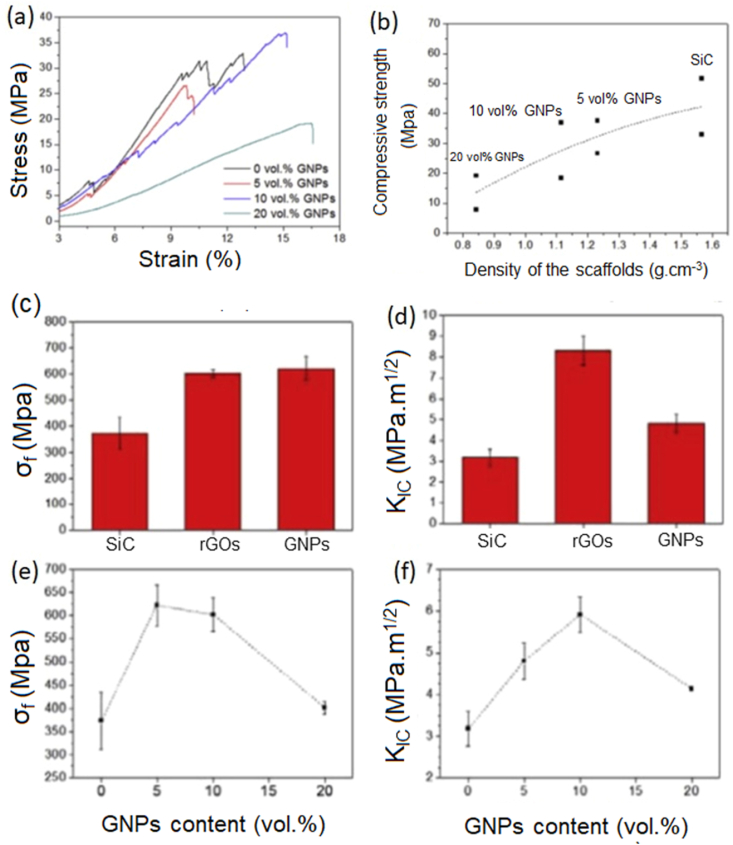
(a) Stress-strain curve of SiC-GNP composite with varying percentage of GNPs, Compressive strength of SiC-GNP composites showing the variation of compressive strength with the density of the scaffold [114]. (c) and (e) Flexural strength, σ, and d) and f) fracture toughness, K_IC_, for SiC-GNP composites: (c) and (d) containing a fixed amount (5 vol%) of different graphene sources, and (e) and (f) varying the GNPs content [106] (Reprinted with permission from Elsevier).

The optimization of the structural performance of porous nanocomposites can be greatly enhanced by the fastening mechanism of GNP to the ceramics. This impact is particularly emphasized by the substantial specific surface area of the three-dimensional GNPs produced by the process of three-dimensional printing [121]. The role of GNP as a nano-reinforcement is manifested through many methods that enhance strength and toughness, including pull-out, interface debonding, and fracture deflection. The influence of the dispersion state of GNP in the ceramic matrix on the structural performance is considered to be a significant factor. In order to mitigate the stress concentration resulting from the clustered arrangement of graphene, which arises from its agglomeration and stacking, it is imperative to take into account the distribution state of GNPs within the ceramic matrix. Appropriate dispersants may be employed to get homogeneous mixing of GNPs, including mechanisms such as electrostatic repulsion and steric interruption. These mechanisms further facilitate the uniform blending of GNP and ceramic powder in the direct mixing approach. Furthermore, the utilization of in-situ creation of ceramic matrix within GNPs is commonly regarded as a method to achieve a consistent and even dispersion. The considerable specific surface area of GNP facilitates the formation of the ceramic matrix, while the interconnected structure resulting from the even distribution of GNPs also enables the diffusion of ceramic precursors. The limited structural uses of 3D-printed GNP-reinforced CMCs can be attributed to the low densification degree, which serves as a significant factor. In contrast to traditional continuous fiber-reinforced CMCs, the extreme porosity of 3-D GNP poses challenges in effectively filling the tiny pores with the injected ceramic matrix. The transmission route to the interior of the structure will be progressively obstructed by the ceramic matrix, resulting in the formation of discernible micropores and a loosely packed arrangement of GNPs [122,123]. Hence, an examination of the densification process of CMCs reinforced with 3-D printed graphene is also undertaken. The fabrication of rGO-Al_2_O_3_ composites with changeable density was carried out by Gil et al. [124] using a combination of 3-D printing and a thermal reduction treatment technique. The development of a completely dense microstructure with fine grains of Al_2_O_3_, in which GNP layers are equally dispersed and trapped between the Al_2_O_3_ grains, is determined by the GO concentration in printable inks and the sintering temperature. Enhancing the volume fraction of graphene is also perceived as a viable approach to attain densification, whereby the matrix may more readily occupy a reduced number of holes. Additionally, it is worthwhile to acquire knowledge and investigate novel techniques, such as the densification process facilitated by the implementation of external pressure and temperature. The fast densification of nano-scaled ceramic powders is achieved by eutectic crystallization under conditions of high temperature or high pressure. Hence, the integration of nano element and matrix materials characterized by high-density and close contact has been identified as a viable approach to address the hands-on implications of utilizing CMCs in structural applications. The SEM surface morphology of the fracture surface of 3-D printed graphene-ceramic composite and the mechanical properties of such composites are exhibited in Fig. 4, Fig. 5 respectively.

### 3.3 3-D printed graphene-metal composite

Metal matrix composites (MMCs) are extensively employed in many industries such as equipment manufacturing, aerospace, and automotive sectors due to their remarkable characteristics including high strength-to-weight ratio, and favorable ductility [[125], [126], [127], [128], [129], [130]]. In the realm of novel extreme application fields, particularly in challenging atmospheres characterized by extreme-high temperatures and greater electromagnetic radiation, there is a need to enhance the mechanical stability in terms of specific strength, specific modulus, corrosion resistance, and thermal conductivity of MMCs. However, conventional methods of reinforcement, such as elemental input, heat treatment, and plastic deformation, have encountered limitations in further enhancing the structural performance [95,[131], [132], [133], [134], [135]]. The large specific surface area of GNP provides significant benefits in terms of reinforcement efficiency when compared to standard reinforcements. These advantages are observed in several aspects, including the mechanical and physical characteristics of nanocomposites. In the current scenario, the utilization of graphene's ultra-low density has the potential for the fabrication of MMCs that are both lightweight and possess great strength. Graphene, when used as a nano-reinforcement, has the capability to enhance the refinement of crystal grains, impede the movement of dislocations, and facilitate load transmission inside nanocomposites. Consequently, this leads to a substantial enhancement in the strength and toughness of the nanocomposites [[136], [137], [138], [139], [140]]. The research conducted by Lin et al. [136] involved the fabrication of iron matrix composites augmented with single-layer graphene oxide (GO) powders via laser sintering. The incorporation of 2 wt% GO into the iron matrix resulted in a significant increase of 93.5% in the surface microhardness of the composites. Additionally, the integration of GO also led to an improvement in the three-point bending fatigue life of the composites [136]. Zhang et al. [141] have put out a method of in-situ processing for fabricating copper-based composites that are reinforced by an intermittent three-dimensional GNP-like network. The resulting nanocomposites have demonstrated enhanced strength and better ductility in comparison to the unreinforced copper [141]. The establishment of a strong interfacial connection between GNP and the metallic base is a crucial prerequisite for achieving structural superiority in MMCs.

In recent times, the fabrication of metal matrix composites reinforced with GNP has primarily involved techniques such as molten process, powder metallurgy, and electrodeposition. These methods offer a relatively controllable and straightforward process for synthesizing composites. However, they also possess certain drawbacks, including inadequate wettability between GNP and the metal interface, insufficient dispersion of GNPs within the matrix, and susceptibility to interfacial reactions [[142], [143], [144], [145]]. Furthermore, it is important to note the significant disparity in density between GNP and MMCs. This discrepancy poses a limitation on the densification process of these composites, hence compromising the optimizing impact of graphene on their structural performance. Metal 3-D printing encompasses many technologies such as selective laser melting (SLM), selective electron beam melting (SEBM), and selective laser sintering (SLS). These techniques enable localized rapid melting and quick cooling processes [[146], [147], [148]]. During the molding process of MMC, the achievement of a high vacuum state facilitates the rapid attainment of a dense state, all the while preserving the structural integrity of the GNPs. Additionally, the 3-D graphene structure that has been produced has a substantial specific surface area and an organized improved network. As a result, the structural stability of MMCs may be greatly boosted, as supported by previous studies [[149], [150], [151]]. The fabrication of bulk GNP-reinforced aluminum (GNP-Al) matrix nanocomposites was conducted by Hu and his colleagues using laser 3-D printing (SLM) [152]. These nanocomposites were fabricated by preparing a combination of GNP and aluminum powders using ball milling, using different weight ratios of GNPs. Subsequently, the mixture was sintered through SLM technique. The SEM and EDS analysis indicated the homogeneous dispersion of GNPs in the Al matrix. Besides, it was observed that the dispersed graphene was in the form of layered and spherical structures, phenomena that can be explained by the presence of robust Van-der-Waals forces and π-π interactions. The formation of agglomerates may be attributed to the presence of robust Van-der-Waals forces and π-π interactions, resulting in a reduction in the contact area between GNPs and aluminum. This reduction in contact area plays a significant role in retarding the response and influencing the structural properties of composites reinforced with GNPs. The elevated temperature used in the laser 3-D printing procedure surpasses the oxidation temperature of graphene, which is 500 °C. Consequently, the thermal buildup leads to an augmentation in the defects present in GNPs. Additionally, the synthesis of the Al_4_C_3_ phase was achieved by the interaction between graphene and aluminum. It is worth noting that the incorporation of a specific quantity of Al_4_C_3_ had a beneficial impact on the strengthening mechanism of the resulting nanocomposites [153,154]. Consequently, the nano-hardness values of pure aluminum (Al) and aluminum specimens with 2.5 wt% of GNP (GNP-Al) were determined to be 1.04 GPa and 1.77 GPa, respectively. Additionally, it is demonstrated that the GNP-Al specimens with 2.5 wt% had significantly smaller penetration depth into the surface when subjected to the same load. It is important to acknowledge that the hardness of the GNP-Al nanocomposite exhibits a reduction as the graphene concentration increases. This phenomenon was attributed to the fact that the out-of-plane strength of multilayer graphene is considerably lower compared to its in-plane characteristics [155]. Moreover, it was observed that the inclusion of 2.5 wt% GNP in the GNP-Al composite resulted in a significant increase of 75.3% in the Vickers hardness value when compared to unreinforced aluminum. The maximum recorded value of Vickers hardness was 66.6 HV [152]. The enhanced structural ability of aluminum matrix composites augmented with GNPs can be ascribed to the inherent mechanical characteristics and the interface condition between the matrix and GNPs [152].

**Fig. 6:**
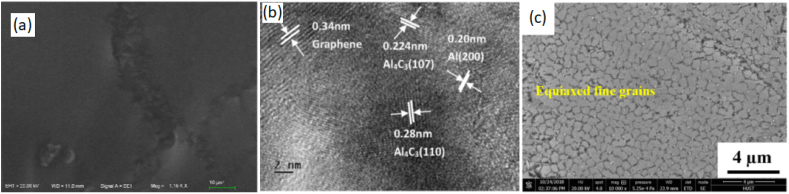
(a) SEM surface morphology of 3D printed 2.5% GNP-Al composite showing the flat surface without containing pores [152] (b) HRTEM image of GNP-Al composites showing the interface pattern of graphene, Al, aluminum carbide [152] (c) SEM surface morphology of 3D printed 0.5% RGo-S136 composite showing the achievement of equiaxed fine grains [157] (Reprinted with permission from Elsevier).

**Fig. 7:**
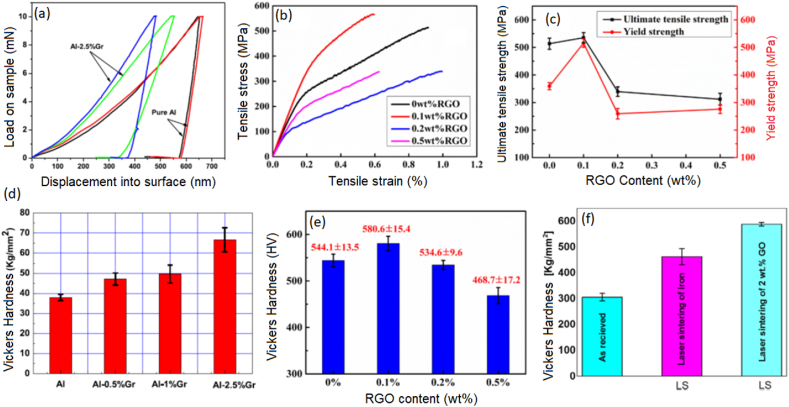
(a) Load-displacement curves of 3-D printed Al and 2.5 wt% GNP-Al composites [152] (b) Stress-strain curve of the rGO/S136 composites with different percentages of rGO [157] (c) relationships of the ultimate tensile strength and yield strength with different rGO contents [157], Vickers hardness value of (d) GNP-Al composite [152] (e) rGO-S136 composite [157] and (f) GO- iron composite [136] (Reprinted with permission from Elsevier).

Shi et al. [156] utilized the design flexibility and layer stacking capability of 3-D printing to produce Ni_3_Al MMCs reinforced with GNPs in order to enhance wear and friction properties. This was achieved through the process of laser melting deposition (LMD), where each individual layer of the composite contained varying amounts of GNPs [156]. The weight percentages of 0–1.5 wt% GNPs were introduced in the first to the fourth layer. The polished surface exhibited no notable fractures or flaws. Upon conducting metallographic corrosion and subsequently magnifying the microstructures in localized regions, varying grains including granular, columnar, and cellular grains with different grain sizes were seen with an increasing concentration of GNPs. The observed variation in hardness between the first to fourth layer of this composite can be attributed to this disparity. The insertion of GNPs into the Ni_3_Al matrix resulted in an increase in the average hardness values from 557.2 HV (0 wt%) to 642.1 HV (1.5 wt%). This improvement in hardness indicates an enhancement in the mechanical capabilities of the material. The observed increase in hardness of GNP-Ni_3_Al containing 1.5 wt% GNPs can be due to the refining of grains and microstructure. The presence of a distinct tribo and transition layers in the cross-section of the wear scar of GNP-Ni_3_Al nanocomposite can be seen and fine GNPs, with a thickness of 3.87 μm, are observed to be deposited on the worn surface of the tribo-layer during the sliding and squeezing process. This phenomenon leads to the formation of a compact and uniform protective layer, thereby enhancing the wear resistance of the material. The enhanced wear resistance of the GNP-Ni_3_Al nanocomposite can be attributed to the significant increase in surface hardness resulting from the incorporation of GNPs. The SEM surface morphology of different 3D printed graphene-reinforced metallic composites and the mechanical properties of such composites have been illustrated in Fig. 6, Fig. 7 respectively. Wen et al. [157] stated the production of reduced graphene oxide (rGO)-S136 MMCs by the integration of liquid deposition and SLM process. The presence of rGO in the metallic matrix has been found to have a significant impact on the dispersion of grain boundaries and grain development. This phenomenon has led to notable enhancements in both yield strength and ultimate tensile strength. Hence, the manipulation of the rGO content in rGO-S136 nanocomposites allows for the customization of grain sizes, crystallographic textures, phase compositions, and mechanical characteristics. The composites exhibit a notable pattern in their tensile strength and yield strength, characterized by an initial increase followed by a subsequent decrease. Notably, when the composites include 0.1 wt% rGO content, the maximum values observed for tensile strength and yield strength are 535.3 MPa and 515.8 MPa, respectively. Moreover, when subjected to a laser beam with a temperature over 1000 °C, it is possible for rGO to undergo a partial reaction with the S136 matrix, resulting in the loss of its distinctive strengthening characteristics. Wang et al. [158] conducted a study in which they produced nanocomposites of GNPs-reinforced Inconel 718 by the process of SLM. The results of their study indicated the formation of a strong link between the GNPs and the matrix. The inclusion of GNPs in the material resulted in enhancements in both ultimate tensile strength and elastic modulus. These improvements can be attributed to the mechanisms of load transfer and dislocation hindrance. In brief, the utilization of additive manufacturing techniques for the production of MMCs reinforced with GNPs allows for fine regulation of the graphene concentration, hence exerting a notable influence on the dimensions of the metal crystal grains and the resulting structural characteristics. Additionally, the building process offers a streamlined approach to designing metal matrix composites [159]. Nevertheless, it is important to acknowledge the dispersion level of GNPs inside the metal, as well as the underlying interfacial attachment mechanism between GNPs and the metal matrix. The achievement of homogeneous distribution and strong alignment of GNPs inside the matrix is crucial for the fabrication of structural composites with superior performance. Additionally, the interaction between GNP and the metal matrix at the interface plays a significant role in determining the structural properties. Moreover, it is imperative to reduce the detrimental effects on the GNPs during the high-temperature phase of the metal 3-D printing process. In several industries such as aircraft, light vehicles, and electronic equipment, the utilization of 3-D printed GNP-reinforced MMCs has proven to be advantageous. These composites can serve as structural components or integrated devices that combine both structural and functional properties.

### 3.4 Other 3D-printed graphene-reinforced structural composites

Graphene exhibits significant potential for various applications, such as drug delivery, biological sensing, tumor treatment, and tissue engineering, primarily as a nano-filler for structural nanocomposites in bone repair implants [[160], [161], [162], [163], [164], [165]]. This is attributed to its non-toxic, extensive specific surface area, and remarkable compatibility with biological systems. The inherent load-carrying ability and flexibility of GNP render nanocomposites make it very promising for advancements in load-carrying capacity and structural fixation of bio-structures [[166], [167], [168], [169], [170]]. Based on these factors, together with the repeatability and design capabilities of 3-D printing, the use of GNPs in the domains of bone regeneration, tissue healing, and organ duplication is significantly broadened [171,172]. The research conducted by Jakus et al. [173] involved the development of intricate and diverse structures using printable composite inks consisting of graphene and hydroxyapatite (referred to as Hyperelastic Bone or HB). These constructions exhibited distinctive mechanical characteristics characterized by non-linear behavior, making them well-suited for the fabrication of mechanically graded tissues [173]. The comparable surface characteristics of hyperelastic bone with 3-D graphene (HB-3DG) and monolithic 3-D graphene indicate that the extensive properties and functionalities of 3-D graphene can be expanded to HB-3DG. Additionally, the cross-sectional analysis shows that the hydroxyapatite (HA) particles are physically enclosed and uniformly dispersed within the GNPs. In addition, the HB-3DG sample had a porosity value of 21.3%, which closely resembled the porosity of the 3D graphene sample at 13.2%. HB-3DG exhibited a preponderance of a percolating graphene network in relation to its structural features and porosity, akin to that observed in 3-D graphene. Hence, the 3-D graphene network assumed a pivotal function in governing the structural characteristics of hyperelastic bone with 3D-graphene (HB-3DG). Besides, the mechanical properties obtained in this composite including the elastic modulus and strain to failure were seen to be 3 MPa and 80%, respectively. These values were found to be notably lower than those of hyperelastic bone (HB). The compressive load of HB-3DG exhibited a linear rise with strain below 28%. However, during the strain range of 28–45%, HB-3DG underwent a transition from plastic to elastic foam behavior. The slide of graphene nanosheets was impeded in conditions of low stresses due to the contact between the layers and the presence of interspersed HA particles. Subsequently, the associated HA particles would exhibit simultaneous slipping with GNP when subjected to failure stress. The utilization of mixed particle systems has the potential to achieve a distinctive and non-linear mechanical characteristic. The utilization of 3-D printed biocompatible porous GNP scaffolds has been demonstrated as a viable approach for serving as templates to facilitate cell adhesion and proliferation. The incorporation of GNPs into scaffolds can enhance their mechanical qualities and thermal conductivity, as well as promote cell development, according to the inherent characteristics of GNPs. Nosrati et al. [174] successfully produced scaffolds using a hydrogel 3-D printing technique. These scaffolds consisted of a combination of rGO, HA, and gelatin. The compressive modulus of these scaffolds was significantly increased, which was due to the greater interaction at the interface between the HA and rGO phases [174]. Besides, Jakus et al. [175] fabricated biocompatible scaffolds using 3D printing technology. These scaffolds were composed of GNPs and a biodegradable material called polylactide-*co*-glycolide (PLG). By adjusting the volume percent of graphene, the researchers were able to control the elastic modulus and hardness of the scaffolds [175]. Shuai et al. [176] employed the selective laser sintering technique to produce diopside scaffolds, which were further enhanced by the incorporation of 1 wt% graphene [176]. The composites exhibited a significant enhancement in both compressive strength and fracture toughness, with improvements of 102% and 34% respectively. These improvements may be attributed to two main factors: the uniform distribution of GNPs inside the diopside matrix, and the refinement of grain structure. In general, it is evident that the strong correlation between GNP and the active particles serves as the primary catalyst for enhancing structural stability. Additionally, the substantial specific surface area of gGNP contributes to the reinforcement of the bonding state. Moreover, the accurate replication capabilities of 3-D printing in the context of biological tissues and organs provide advantageous prospects for advancing the application potential of GNPs within the realm of tissue engineering.

## 4 Conclusions and future perspective

In recent times, there has been a significant focus among researchers on utilizing graphene as a strengthening medium in composites to enhance their mechanical response and functional characteristics. Additionally, several notable studies have been published on the use of 3-D printing techniques to fabricate three-dimensional graphene structures. The primary goal of this study is to deliver a comprehensive synopsis of the structural implementation of 3-D printed nanocomposites strengthened by GNPs. The analysis primarily encompasses three types of composites: graphene-polymer composites, graphene-ceramic composites, and graphene-metal composites. The present study provides a widespread analysis of the optimization process of GNPs in enhancing the mechanical stability of the nanocomposite structures. Specifically, the focus is on the crucial role played by the comprehensible state between GNP and matrix materials. Furthermore, this study introduces many 3-D printing techniques that are adaptable to diverse composite materials, including direct ink writing (DIW), laser melting deposition (LMD), and selective laser melting (SLM). In light of the aforementioned factors, this study provides a broad assessment of the merits and drawbacks pertaining to the structural efficacy of 3-D printed composites reinforced with GNP. Additionally, it addresses the pressing challenges that require immediate resolution and identifies the key areas of attention for future investigations. In addition to the aforementioned optimized matrix discussed in this article, it is worth noting the structural implementation of GNP-reinforced tissue engineering nanocomposites in the domains of cell production, bone restoration, and bone assembly rebuilding. These applications are of particular interest due to the involvement of the biocompatibility of GNPs. Hence, it can be inferred that GNPs possess the prospective for application in composite domains, hence enhancing structural performance by virtue of their inherent mechanical qualities and substantial specific surface area. Despite the considerable promise demonstrated by 3D-printed composites reinforced with graphene in enhancing structural performance and its use in critical domains, further research is necessary to address the following limitations associated with graphene and the manufacturing procedures employed for these composites.

a) The inks employed in the realm of 3-D printing predominantly rely on graphene or graphene oxide as primary constituents. However, the existing techniques for their production pose challenges in terms of achieving precise control over the dimensions and layer count of graphene. Consequently, this lack of control gives rise to unmanageable structural imperfections and contamination within the graphene material.
b) The presence of a robust van-der-waals force between the sheets of GNPs facilitates invariable agglomeration. This phenomenon leads to the non-homogeneous dispersion of GNP within the matrix and results in concentrating stress in the clustered reinforcement region in the nanocomposites. Therefore, the structural strengthening parameters of GNP-reinforced composites are affected and potentially weakened [177,178].
c) The insertion of suitable solvents and hydrophilic compounds on the surface of GNPs is thought to be an efficient technique to produce consistent and stable distribution [31,179]. The procedure effectively prevents the accumulation and clustering of GNPs by utilizing electrostatic repulsion and steric restriction processes. Additionally, it successfully produces printable inks based on graphene that exhibit the expected rheological properties.
d) Graphene oxide (GO) can serve as a starting material owing to its dispersibility and substantial dimension. Thus, chemical decomposition or thermal lessening methods are commonly employed to eliminate functional groups containing oxygen, thereby enhancing the structural strengthening effects in nanocomposites [33,180].
e) Furthermore, the ability to control and precision of the 3-D printing process for the building of composite framework still needs to be developed. Currently, the utilization of superior-resolution printing techniques has been employed in the fabrication of precise mechanical components and microelectronic elements. This micro-scale refinement technique has the potential to yield unforeseen enhancements in the structural properties of nanocomposites, akin to the design principles employed in metamaterials.
f) The utilization of high-resolution 3-D printing in the development of low-scale structural design allows for the incorporation of distinctive structural attributes in nanocomposites. These attributes include qualities such as lightweights, desirable poisson's ratio, and greater elasticity.
g) The utilization of micro-scale printing techniques has emerged as a prominent trajectory in the area of tissue engineering structural nanocomposites. This advancement holds the potential to greatly broaden the scope of applications, encompassing bone scaffold assemblies and even cell constructions for in vitro replacement. By leveraging the advantageous bio-compliance and mechanical properties of GNPs, this technology enables the realization of enhanced functionalities.
h) Finally, the damage to GNP during the 3-D printing process and the matrix introduction method is also worthy of concern. For instance, the thermal conditions involved in the printing procedure of metal matrix composites reinforced with graphene typically involve elevated temperatures during the stages of melting and sintering. Similarly, the temperature requirements for the induction and densification process of the ceramic matrix in GNP-ceramic nanocomposites also surpass 1000 °C. Consequently, the elevated temperature would induce permanent harm and generate a significant quantity of imperfections inside the graphene nanosheets, substantially impacting both the mechanical characteristics and the efficacy of structural optimization.
i) The implementation of reducing the reaction temperature and including a protective covering on the surface of GNPs has been employed as a means of safeguarding them. The various mechanisms of synthesis and densification of matrix materials have the potential to decrease the temperature throughout the reaction process. One possible approach to introducing the ceramic matrix is by the cracking of the precursor, as opposed to the in-situ growth method using chemical vapor deposition (CVD). This alternative method has the potential to lower the response temperature to a value lower than 1000 °C. In addition, the implementation of high-temperature and oxidation-resistant coatings on GNPs has the potential to create a proficient protective layer. Examples of such coatings are Boron nitride (BN) and SiC interfaces, which have the capability to provide effective protection in ultra-high temperatures.

In general, the prospects and possibilities of utilizing 3-D-printed GNP-reinforced nanocomposites in structural applications are highly promising. The distinctive structural features of GNPs, including their large specific surface area and exceptional mechanical strength, are attributed to their unique 2-D honeycomb lattice single-atom layer assembly. The utilization of this material as an optimal nanofiller for enhancing the structural properties of nanocomposites is feasible. The adjustable design of GNP's physical characteristics, together with its distribution state in the matrix materials and comprehensible state with the matrix, merits careful consideration [181,182]. More significantly, as a new material and creative preparation technique employed in structural nanocomposites, GNP, and 3-D printing should be studied extensively, especially the optimal mechanism and evolution process.

## Data availability statement

Data will be made available on request.

## Prime novelty statement

This paper comprehensively highlights how the incorporation of graphene and the 3-D printing process strengthen multiscale structural composites. Although various issues and prospects of the 3D printing process or the materials prepared by 3-D printing were addressed in a number of review articles, none of them comprehensively highlighted how the properties change and how the printing process improves the mechanical qualities that can be useful for structures at micro and macro levels. In addition, there has been no previous review on the implementation of composites in the biological structure. Therefore, the prime novelty of this article is to highlight the current research results on 3-D printed nanocomposites for advanced micro- and macro-level structures, including those in the biological domain. In this review article, different 3D printing techniques have been discussed, and the application and utilization of these techniques in improving the structural qualities of different types of composite materials have been highlighted. Additionally, this review article anticipates analyzing the current obstacles and potential future advancements in this rapidly expanding domain.

## CRediT authorship contribution statement

**AKM Asif Iqbal:** Writing – review & editing, Supervision, Project administration, Conceptualization, Validation. **Clement Stefano Harcen:** Writing – original draft, Resources, Investigation, Formal analysis, Data curation. **Mainul Haque:** Visualization, Project administration, Writing – review & editing.

## Declaration of competing interest

The authors declare that they have no known competing financial interests or personal relationships that could have appeared to influence the work reported in this paper.

## References

[1] Diamanti K., Soutis C. (2010). Structural health monitoring techniques for aircraft composite structures. Prog. Aero. Sci..

[2] Verpoest I., Lomov S.V. (2005). Virtual textile composites software WiseTex: integration with micro-mechanical, permeability and structural analysis. Compos. Sci. Technol..

[3] Snead L.L., Nozawa T., Ferraris M., Katoh Y., Shinavski R., Sawan M. (2011). Silicon carbide composites as fusion power reactor structural materials. J. Nucl. Mater..

[4] Hesabi Z.R., Simchi A., Reihani S.M.S. (2006). Structural evolution during mechanical milling of nanometric and micrometric Al2O3 reinforced Al matrix composites. Mater. Sci. Eng..

[5] Safri S.N.A., Sultan M.T.H., Jawaid M., Jayakrishna K. (2018). Impact behaviour of hybrid composites for structural applications: a review. Compos. B Eng..

[6] Clarissa W.H.Y., Chia C.H., Zakaria S., Evyan Y.C.Y. (2022). Recent advancement in 3-D printing: nanocomposites with added functionality. Progress in Additive Manufacturing.

[7] Ngo T.D., Kashani A., Imbalzano G., Nguyen K.T.Q., Hui D. (2018). Additive manufacturing (3D printing): a review of materials, methods, applications and challenges. Compos. B Eng..

[8] Au A.K., Huynh W., Horowitz L.F., Folch A. (2016). Mikrofluidik aus dem 3D‐Drucker. Angew. Chem..

[9] Compton B.G., Lewis J.A. (2014). 3D-printing of lightweight cellular composites. Adv. Mater..

[10] Huang S.H., Liu P., Mokasdar A., Hou L. (2013). Additive manufacturing and its societal impact: a literature review. Int. J. Adv. Manuf. Technol..

[11] Gardner J.M., Sauti G., Kim J.W., Cano R.J., Wincheski R.A., Stelter C.J. (2016). 3-D printing of multifunctional carbon nanotube yarn reinforced components. Addit. Manuf..

[12] Yao Y., Fu K.K., Yan C., Dai J., Chen Y., Wang Y. (2016). Three-dimensional printable high-temperature and high-rate heaters. ACS Nano.

[13] Sun K., Wei T.S., Ahn B.Y., Seo J.Y., Dillon S.J., Lewis J.A. (2013). 3D printing of interdigitated Li-ion microbattery architectures. Adv. Mater..

[14] Huang K., Dong S., Yang J., Yan J., Xue Y., You X. (2019). Three-dimensional printing of a tunable graphene-based elastomer for strain sensors with ultrahigh sensitivity. Carbon N Y.

[15] Gnanasekaran K., Heijmans T., van Bennekom S., Woldhuis H., Wijnia S., de With G. (2017). 3D printing of CNT- and graphene-based conductive polymer nanocomposites by fused deposition modeling. Appl. Mater. Today.

[16] Campbell T.A., Ivanova O.S. (2013). 3D printing of multifunctional nanocomposites. Nano Today.

[17] Trebbin M., Steinhauser D., Perlich J., Buffet A., Roth S.V., Zimmermann W. (2013). Anisotropic particles align perpendicular to the flow direction in narrow microchannels. Proc Natl Acad Sci U S A.

[18] Heller B.P., Smith D.E., Jack D.A. (2016). Effects of extrudate swell and nozzle geometry on fiber orientation in Fused Filament Fabrication nozzle flow. Addit. Manuf..

[19] An J., Teoh J.E.M., Suntornnond R., Chua C.K. (2015). Design and 3D printing of scaffolds and tissues. Engineering.

[20] Fu K., Yao Y., Dai J., Hu L. (2017). Progress in 3D printing of carbon materials for energy-related applications. Adv. Mater..

[21] Yuan C.Z., Gao B., Shen L.F., Yang S.D., Hao L., Lu X.J. (2011). Hierarchically structured carbon-based composites: design, synthesis and their application in electrochemical capacitors. Nanoscale.

[22] Bose S., Kuila T., Mishra A.K., Rajasekar R., Kim N.H., Lee J.H. (2012). Carbon-based nanostructured materials and their composites as supercapacitor electrodes. J. Mater. Chem..

[23] Geim AK, Novoselov KS. The Rise of Graphene. n.d. 10.1038/nmat1849 17330084

[24] Stankovich S., Dikin D.A., Dommett G.H.B., Kohlhaas K.M., Zimney E.J., Stach E.A. (2006). Graphene-based composite materials. Nature.

[25] Wei X., Li D., Jiang W., Gu Z., Wang X., Zhang Z. (2015). 3D printable graphene composite. Sci. Rep..

[26] Zhu Y., Murali S., Cai W., Li X., Suk J.W., Potts J.R. (2010). Graphene and graphene oxide: synthesis, properties, and applications. Adv. Mater..

[27] You X., Yang J., Dong S. (2021). Structural and functional applications of 3D-printed graphene-based architectures. J. Mater. Sci..

[28] Medhekar N.V., Ramasubramaniam A., Ruoff R.S., Shenoy V.B. (2010). Hydrogen bond networks in graphene oxide composite paper: structure and mechanical properties. ACS Nano.

[29] Wang L., Li Y., Han Z., Chen L., Qian B., Jiang X. (2013). Composite structure and properties of Mn3O4/graphene oxide and Mn3O4/graphene. J Mater Chem A Mater.

[30] Huang L., Li C., Yuan W., Shi G. (2013). Strong composite films with layered structures prepared by casting silk fibroin-graphene oxide hydrogels. Nanoscale.

[31] You X., Yang J., Wang M., Wang H., Gao L., Dong S. (2020). Interconnected graphene scaffolds for functional gas sensors with tunable sensitivity. J. Mater. Sci. Technol..

[32] Sha J., Li Y., Villegas Salvatierra R., Wang T., Dong P., Ji Y. (2017). Three-dimensional printed graphene foams. ACS Nano.

[33] Zhang Q., Zhang F., Medarametla S.P., Li H., Zhou C., Lin D. (2016). 3D printing of graphene aerogels. Small.

[34] Zhu C., Liu T., Qian F., Han T.Y.J., Duoss E.B., Kuntz J.D. (2016). Supercapacitors based on three-dimensional hierarchical graphene aerogels with periodic macropores. Nano Lett..

[35] Huang K., Yang J., Dong S., Feng Q., Zhang X., Ding Y. (2018). Anisotropy of graphene scaffolds assembled by three-dimensional printing. Carbon N Y.

[36] Wang X., Jiang M., Zhou Z., Gou J., Hui D. (2017). 3D printing of polymer matrix composites: a review and prospective. Compos. B Eng..

[37] Mohamed O.A., Masood S.H., Bhowmik J.L. (2015). Optimization of fused deposition modeling process parameters: a review of current research and future prospects. Adv. Manuf..

[38] Sood A.K., Ohdar R.K., Mahapatra S.S. (2010). Parametric appraisal of mechanical property of fused deposition modelling processed parts. Mater. Des..

[39] Chohan J.S., Singh R., Boparai K.S., Penna R., Fraternali F. (2017). Dimensional accuracy analysis of coupled fused deposition modeling and vapour smoothing operations for biomedical applications. Compos. B Eng..

[40] Parandoush P., Lin D. (2017). A review on additive manufacturing of polymer-fiber composites. Compos. Struct..

[41] Wang X., Jiang M., Zhou Z., Gou J., Hui D. (2017). 3D printing of polymer matrix composites: a review and prospective. Compos. B Eng..

[42] Dou R., Wang T., Guo Y., Derby B. (2011). Ink‐jet printing of zirconia: coffee staining and line stability. J. Am. Ceram. Soc..

[43] Travitzky N., Bonet A., Dermeik B., Fey T., Filbert‐Demut I., Schlier L. (2014). Additive manufacturing of ceramic‐based materials. Adv. Eng. Mater..

[44] Khoshnevis B. (2004). Automated construction by contour crafting - related robotics and information technologies. Autom Constr.

[45] Melchels F.P.W., Feijen J., Grijpma D.W. (2010). A review on stereolithography and its applications in biomedical engineering. Biomaterials.

[46] Eckel Z.C., Zhou C., Martin J.H., Jacobsen A.J., Carter W.B., Schaedler T.A. (2016). Additive manufacturing of polymer-derived ceramics. Science (1979).

[47] Manapat J.Z., Chen Q., Ye P., Advincula R.C. (2017). 3D printing of polymer nanocomposites via stereolithography. Macromol. Mater. Eng..

[48] Utela B., Storti D., Anderson R., Ganter M. (2008). A review of process development steps for new material systems in three dimensional printing (3DP). J. Manuf. Process..

[49] Lee H., Lim C.H.J., Low M.J., Tham N., Murukeshan V.M., Kim Y.J. (2017). Lasers in additive manufacturing: a review. International Journal of Precision Engineering and Manufacturing - Green Technology.

[50] Yap C.Y., Chua C.K., Dong Z.L., Liu Z.H., Zhang D.Q., Loh L.E. (2015). Review of selective laser melting: materials and applications. Appl. Phys. Rev..

[51] Gibson I., Rosen D., Stucker B. (2015).

[52] Ngo T.D., Kashani A., Imbalzano G., Nguyen K.T.Q., Hui D. (2018). Additive manufacturing (3D printing): a review of materials, methods, applications and challenges. Compos. B Eng..

[53] Williams S.W., Martina F., Addison A.C., Ding J., Pardal G., Colegrove P. (2016). Wire + Arc Additive manufacturing. Mater. Sci. Technol..

[54] Gibson I., Rosen D., Stucker B. (2015).

[55] Li J., Monaghan T., Nguyen T.T., Kay R.W., Friel R.J., Harris R.A. (2017). Multifunctional metal matrix composites with embedded printed electrical materials fabricated by ultrasonic additive manufacturing. Compos. B Eng..

[56] Hahnlen R., Dapino M.J. (2014). NiTi–Al interface strength in ultrasonic additive manufacturing composites. Compos. B Eng..

[57] Hehr A., Dapino M.J. (2015). Interfacial shear strength estimates of NiTi–Al matrix composites fabricated via ultrasonic additive manufacturing. Compos. B Eng..

[58] Kazemian A., Yuan X., Cochran E., Khoshnevis B. (2017). Cementitious materials for construction-scale 3D printing: laboratory testing of fresh printing mixture. Constr Build Mater.

[59] Kusaseh N.M., Nuruzzaman D.M., Ismail N.M., Hamedon Z., Azhari A., Iqbal A.K.M.A. (2018). Flexure and impact properties of glass fiber reinforced nylon 6-polypropylene composites. IOP Conf. Ser. Mater. Sci. Eng..

[60] Asif Iqbal A., Arai Y. (2015). Study on low-cycle fatigue behavior of cast hybrid metal matrix composites. Int. J. Automot. Mech. Eng..

[61] Nuruzzaman D.M., Kusaseh N., Ismail N.M., Iqbal A.K.M.A., Rahman M.M., Azhari A. (2020). Influence of glass fiber content on tensile properties of polyamide-polypropylene based polymer blend composites. Mater Today Proc.

[62] Iqbal A.A., Arai Y., Araki W. (2022). Influence of residual stress on the fatigue crack growth mechanism in the Al-Alloy/Hybrid MMC Bi-material. J. Fail. Anal. Prev..

[63] Iqbal A.A., Ismail N.B. (2022). Mechanical properties and corrosion behavior of silica nanoparticle reinforced magnesium nanocomposite for bio-implant application. Materials.

[64] Das B., Eswar Prasad K., Ramamurty U., Rao C.N.R. (2009). Nano-indentation studies on polymer matrix composites reinforced by few-layer graphene. Nanotechnology.

[65] Ji X., Xu Y., Zhang W., Cui L., Liu J. (2016). Review of functionalization, structure and properties of graphene/polymer composite fibers. Compos Part A Appl Sci Manuf.

[66] Du J., Cheng H.M. (2012). The fabrication, properties, and uses of graphene/polymer composites. Macromol. Chem. Phys..

[67] Zhao Y.H., Wu Z.K., Bai S.L. (2015). Study on thermal properties of graphene foam/graphene sheets filled polymer composites. Compos Part A Appl Sci Manuf.

[68] Wang M., Duan X., Xu Y., Duan X. (2016). Functional three-dimensional graphene/polymer composites. ACS Nano.

[69] Wajid A.S., Das S., Irin F., Ahmed H.S.T., Shelburne J.L., Parviz D. (2012). Polymer-stabilized graphene dispersions at high concentrations in organic solvents for composite production. Carbon N Y.

[70] Villar-Rodil S., Paredes J.I., Martínez-Alonso A., Tascón J.M.D. (2009). Preparation of graphene dispersions and graphene-polymer composites in organic media. J. Mater. Chem..

[71] Ma J., Meng Q., Zaman I., Zhu S., Michelmore A., Kawashima N. (2014). Development of polymer composites using modified, high-structural integrity graphene platelets. Compos. Sci. Technol..

[72] Bora C., Bharali P., Baglari S., Dolui S.K., Konwar B.K. (2013). Strong and conductive reduced graphene oxide/polyester resin composite films with improved mechanical strength, thermal stability and its antibacterial activity. Compos. Sci. Technol..

[73] Vadukumpully S., Paul J., Mahanta N., Valiyaveettil S. (2011). Flexible conductive graphene/poly(vinyl chloride) composite thin films with high mechanical strength and thermal stability. Carbon N Y.

[74] De Leon A.C., Chen Q., Palaganas N.B., Palaganas J.O., Manapat J., Advincula R.C. (2016). High performance polymer nanocomposites for additive manufacturing applications. React. Funct. Polym..

[75] Markandan K., Lai C.Q. (2020). Enhanced mechanical properties of 3D printed graphene-polymer composite lattices at very low graphene concentrations. Compos Part A Appl Sci Manuf.

[76] Lin D., Jin S., Zhang F., Wang C., Wang Y., Zhou C. (2015). 3D stereolithography printing of graphene oxide reinforced complex architectures. Nanotechnology.

[77] Qin Z, Jung GS, Kang MJ, Buehler MJ. The Mechanics and Design of a Lightweight Three-Dimensional Graphene Assembly. n.d. 10.1126/sciadv.1601536 PMC5218516 28070559

[78] Liang Z., Pei Y., Chen C., Jiang B., Yao Y., Xie H. (2019). General, vertical, three-dimensional printing of two-dimensional materials with multiscale alignment. ACS Nano.

[79] Farahani R.D., Dubé M., Therriault D. (2016). Three-dimensional printing of multifunctional nanocomposites: manufacturing techniques and applications. Adv. Mater..

[80] Lee S., Wajahat M., Kim J.H., Pyo J., Chang W.S., Cho S.H. (2019). Electroless deposition-assisted 3D printing of micro circuitries for structural electronics. ACS Appl. Mater. Interfaces.

[81] Zhong J., Zhou G.X., He P.G., Yang Z.H., Jia D.C. (2017). 3D printing strong and conductive geo-polymer nanocomposite structures modified by graphene oxide. Carbon N Y.

[82] Zhang G., Song D., Jiang J., Li W., Huang H., Yu Z. (2023). Electrically assisted continuous vat photopolymerization 3D printing for fabricating high-performance ordered graphene/polymer composites. Compos. B Eng..

[83] Bustillos J., Montero D., Nautiyal P., Loganathan A., Boesl B., Agarwal A. (2018). Integration of graphene in poly(lactic) acid by 3D printing to develop creep and wear‐resistant hierarchical nanocomposites. Polym. Compos..

[84] Zhang G., Song D., Jiang J., Li W., Huang H., Yu Z. (2023). Electrically assisted continuous vat photopolymerization 3D printing for fabricating high-performance ordered graphene/polymer composites. Compos. B Eng..

[85] Sakib MdN., Asif Iqba A. (2021). Epoxy based nanocomposite material for automotive application- A short review. Int. J. Automot. Mech. Eng..

[86] You X., Yang J., Wang M., Zhou H., Gao L., Hu J. (2020). Novel graphene planar architecture with ultrahigh stretchability and sensitivity. ACS Appl. Mater. Interfaces.

[87] Wang Z., Zhang Q., Yue Y., Xu J., Xu W., Sun X. (2019). 3D printed graphene/polydimethylsiloxane composite for stretchable strain sensor with tunable sensitivity. Nanotechnology.

[88] Spinelli G., Kotsilkova R., Ivanov E., Petrova-Doycheva I., Menseidov D., Georgiev V. (2019). Effects of filament extrusion, 3D printing and hot-pressing on electrical and tensile properties of poly(lactic) acid composites filled with carbon nanotubes and graphene. Nanomaterials.

[89] Kim M., Jeong J.H., Lee J.-Y., Capasso A., Bonaccorso F., Kang S.-H. (2019). Electrically conducting and mechanically strong graphene–polylactic acid composites for 3D printing. ACS Appl. Mater. Interfaces.

[90] Li Z., Wang Z., Gan X., Fu D., Fei G., Xia H. (2017). Selective laser sintering 3D printing: a way to construct 3D electrically conductive segregated network in polymer matrix. Macromol. Mater. Eng..

[91] Lawes S., Riese A., Sun Q., Cheng N., Sun X. (2015). Printing nanostructured carbon for energy storage and conversion applications. Carbon N Y.

[92] Wang J., Liu Y., Fan Z., Wang W., Wang B., Guo Z. (2019). Ink-based 3D printing technologies for graphene-based materials: a review. Adv. Compos. Hybrid Mater..

[93] Manapat J.Z., Mangadlao J.D., Tiu B.D.B., Tritchler G.C., Advincula R.C. (2017). High-strength stereolithographic 3D printed nanocomposites: graphene oxide metastability. ACS Appl. Mater. Interfaces.

[94] Naslain R.R. (2005). SiC-matrix composites: nonbrittle ceramics for thermo-structural application. Int. J. Appl. Ceram. Technol..

[95] Nieto A., Bisht A., Lahiri D., Zhang C., Agarwal A. (2017). Graphene reinforced metal and ceramic matrix composites: a review. Int. Mater. Rev..

[96] Xia Z., Riester L., Curtin W.A., Li H., Sheldon B.W., Liang J. (2004). Direct observation of toughening mechanisms in carbon nanotube ceramic matrix composites. Acta Mater..

[97] Hu J., Dong S., Feng Q., Zhou M., Wang X., Cheng Y. (2014). Tailoring carbon nanotube/matrix interface to optimize mechanical properties of multiscale composites. Carbon N Y.

[98] Hu Z., Dong S., Hu J., Lu B. (2013). Fabrication and properties analysis of Cf–CNT/SiC composite. Ceram. Int..

[99] Mei H., Cheng L. (2009). Comparison of the mechanical hysteresis of carbon/ceramic-matrix composites with different fiber preforms. Carbon N Y.

[100] Baldus P, Jansen M, Sporn D. Ceramic Fibers for Matrix Composites in High-Temperature Engine Applications. n.d. 10.1126/science.285.5428.699 10426985

[101] Hu J., Dong S., Wu B., Zhang X., Wang Z., Zhou H. (2013). Mechanical and thermal properties of Cf/SiC composites reinforced with carbon nanotube grown in situ. Ceram. Int..

[102] Porwal H., Grasso S., Reece M.J. (2013). Review of graphene–ceramic matrix composites. Adv. Appl. Ceram..

[103] Smirnov A., Peretyagin P., Bartolomé J.F. (2019). Processing and mechanical properties of new hierarchical metal-graphene flakes reinforced ceramic matrix composites. J. Eur. Ceram. Soc..

[104] Zhou B.Y., Fan S.J., Fan Y.C., Zheng Q., Zhang X., Jiang W. (2020). Recent progress in ceramic matrix composites reinforced with graphene nanoplatelets. Rare Met..

[105] Tapasztó O., Tapasztó L., Markó M., Kern F., Gadow R., Balázsi C. (2011). Dispersion patterns of graphene and carbon nanotubes in ceramic matrix composites. Chem. Phys. Lett..

[106] Belmonte M., Nistal A., Boutbien P., Román-Manso B., Osendi M.I., Miranzo P. (2016). Toughened and strengthened silicon carbide ceramics by adding graphene-based fillers. Scr Mater.

[107] Walker L.S., Marotto V.R., Rafiee M.A., Koratkar N., Corral E.L. (2011). Toughening in graphene ceramic composites. ACS Nano.

[108] Cao W.Q., Wang X.X., Yuan J., Wang W.Z., Cao M.S. (2015). Temperature dependent microwave absorption of ultrathin graphene composites. J Mater Chem C Mater.

[109] Wan Y.J., Gong L.X., Tang L.C., Wu L Bin, Jiang J.X. (2014). Mechanical properties of epoxy composites filled with silane-functionalized graphene oxide. Compos Part A Appl Sci Manuf.

[110] Song W.L., Cao M.S., Lu M.M., Bi S., Wang C.Y., Liu J. (2014). Flexible graphene/polymer composite films in sandwich structures for effective electromagnetic interference shielding. Carbon N Y.

[111] Meng X., Xu C., Xiao G., Yi M., Zhang Y. (2016). Microstructure and anisotropy of mechanical properties of graphene nanoplate toughened Al2O3-based ceramic composites. Ceram. Int..

[112] Huang Y., Wan C. (2020). Controllable fabrication and multifunctional applications of graphene/ceramic composites. Journal of Advanced Ceramics.

[113] Román-Manso B., Sánchez-González E., Ortiz A.L., Belmonte M., Isabel Osendi M., Miranzo P. (2014). Contact-mechanical properties at pre-creep temperatures of fine-grained graphene/SiC composites prepared in situ by spark-plasma sintering. J. Eur. Ceram. Soc..

[114] Román-Manso B., Figueiredo F.M., Achiaga B., Barea R., Pérez-Coll D., Morelos-Gómez A. (2016). Electrically functional 3D-architectured graphene/SiC composites. Carbon N Y.

[115] Smay J.E., Cesarano J., Lewis J.A. (2002). Colloidal inks for directed assembly of 3-D periodic structures. Langmuir.

[116] Hashemi R., Weng G.J. (2016). A theoretical treatment of graphene nanocomposites with percolation threshold, tunneling-assisted conductivity and microcapacitor effect in AC and DC electrical settings. Carbon N Y.

[117] Wang Y., Shan J.W., Weng G.J. (2015). Percolation threshold and electrical conductivity of graphene-based nanocomposites with filler agglomeration and interfacial tunneling. J. Appl. Phys..

[118] Eom J.H., Kim Y.W., Raju S. (2013). Processing and properties of macroporous silicon carbide ceramics: a review. Journal of Asian Ceramic Societies.

[119] Pierin G., Grotta C., Colombo P., Mattevi C. (2016). Direct Ink Writing of micrometric SiOC ceramic structures using a preceramic polymer. J. Eur. Ceram. Soc..

[120] Huang Y.H., Jiang D.L., Chen Z.M., Liu X.J., Zhang X.F., Liao Z.K. (2018). Fabrication and property of rGO/SiC composite. Wuji Cailiao Xuebao/Journal of Inorganic Materials.

[121] Cheng Y., Lyu Y., Han W., Hu P., Zhou S., Zhang X. (2019). Multiscale toughening of ZrB _2_ ‐SiC‐Graphene@ZrB _2_ ‐SiC dual composite ceramics. J. Am. Ceram. Soc..

[122] Zhou M., Lin T., Huang F., Zhong Y., Wang Z., Tang Y. (2013). Highly conductive porous graphene/ceramic composites for heat transfer and thermal energy storage. Adv. Funct. Mater..

[123] Balázsi K., Furkó M., Liao Z., Gluch J., Medved D., Sedlák R. (2020). Porous sandwich ceramic of layered silicon nitride-zirconia composite with various multilayered graphene content. J. Alloys Compd..

[124] Tubío C.R., Rama A., Gómez M., del Río F., Guitián F., Gil A. (2018). 3D-printed graphene-Al2O3 composites with complex mesoscale architecture. Ceram. Int..

[125] Casati R., Vedani M. (2014). Metal matrix composites reinforced by nano-particles—a review. Metals.

[126] Hashim J., Looney L., Hashmi M.S.J. (1999). Metal matrix composites: production by the stir casting method. J. Mater. Process. Technol..

[127] Kaczmar J.W., Pietrzak K., Włosiński W. (2000). The production and application of metal matrix composite materials. J. Mater. Process. Technol..

[128] Chou T.W., Kelly A., Okura A. (1985). Fibre-reinforced metal-matrix composites. Composites.

[129] Xiong B., Cheng D., Zheng F., Peng F., Zhu Q., Niu Z. (2024). Strengthening mechanisms in graphene reinforced Nb/Nb5Si3 composite. J. Alloys Compd..

[130] Xiong B., Peng F., Chen W., Li C., Zhu Q., Niu Z. (2023). Outstanding strength and toughness in graphene reinforced Nb/Nb5Si3 composites with interfacial nano-phases. J. Mater. Res. Technol..

[131] Shirvanimoghaddam K., Hamim S.U., Karbalaei Akbari M., Fakhrhoseini S.M., Khayyam H., Pakseresht A.H. (2017). Carbon fiber reinforced metal matrix composites: fabrication processes and properties. Compos Part A Appl Sci Manuf.

[132] Qu X., Zhang L., Wu M., Ren S. (2011). Review of metal matrix composites with high thermal conductivity for thermal management applications. Prog. Nat. Sci.: Mater. Int..

[133] Slipenyuk A., Kuprin V., Milman Y., Goncharuk V., Eckert J. (2006). Properties of P/M processed particle reinforced metal matrix composites specified by reinforcement concentration and matrix-to-reinforcement particle size ratio. Acta Mater..

[134] Han X.-H., Wang Q., Park Y.-G., T'Joen C., Sommers A., Jacobi A. (2012). A review of metal foam and metal matrix composites for heat exchangers and heat sinks. Heat Tran. Eng..

[135] Bains P.S., Sidhu S.S., Payal H.S. (2016). Fabrication and machining of metal matrix composites: a review. Mater. Manuf. Process..

[136] Lin D., Richard Liu C., Cheng G.J. (2014). Single-layer graphene oxide reinforced metal matrix composites by laser sintering: microstructure and mechanical property enhancement. Acta Mater..

[137] El-Ghazaly A., Anis G., Salem H.G. (2017). Effect of graphene addition on the mechanical and tribological behavior of nanostructured AA2124 self-lubricating metal matrix composite. Compos Part A Appl Sci Manuf.

[138] Güler Ö., Bağcı N. (2020). A short review on mechanical properties of graphene reinforced metal matrix composites. J. Mater. Res. Technol..

[139] Jin B., Xiong D.-B., Tan Z., Fan G., Guo Q., Su Y. (2019). Enhanced corrosion resistance in metal matrix composites assembled from graphene encapsulated copper nanoflakes. Carbon N Y.

[140] Naseer A., Ahmad F., Aslam M., Guan B.H., Harun W.S.W., Muhamad N. (2019). A review of processing techniques for graphene-reinforced metal matrix composites. Mater. Manuf. Process..

[141] Zhang X., Shi C., Liu E., He F., Ma L., Li Q. (2017). Achieving high strength and high ductility in metal matrix composites reinforced with a discontinuous three-dimensional graphene-like network. Nanoscale.

[142] Boostani A.F., Mousavian R.T., Tahamtan S., Yazdani S., Khosroshahi R.A., Wei D. (2015). Graphene sheets encapsulating SiC nanoparticles: a roadmap towards enhancing tensile ductility of metal matrix composites. Mater. Sci. Eng..

[143] Shin S.E., Choi H.J., Shin J.H., Bae D.H. (2015). Strengthening behavior of few-layered graphene/aluminum composites. Carbon N Y.

[144] Yan S.J., Dai S.L., Zhang X.Y., Yang C., Hong Q.H., Chen J.Z. (2014). Investigating aluminum alloy reinforced by graphene nanoflakes. Mater. Sci. Eng..

[145] Pavithra C.L.P., Sarada B.V., Rajulapati K.V., Rao T.N., Sundararajan G. (2014). A new electrochemical approach for the synthesis of copper-graphene nanocomposite foils with high hardness. Sci. Rep..

[146] Olakanmi E.O., Cochrane R.F., Dalgarno K.W. (2015). A review on selective laser sintering/melting (SLS/SLM) of aluminium alloy powders: processing, microstructure, and properties. Prog. Mater. Sci..

[147] Sercombe T.B., Li X. (2016). Selective laser melting of aluminium and aluminium metal matrix composites: review. Mater. Technol..

[148] Gu D.D., Meiners W., Wissenbach K., Poprawe R. (2012). Laser additive manufacturing of metallic components: materials, processes and mechanisms. Int. Mater. Rev..

[149] Dong M., Zhou W., Kamata K., Nomura N. (2020). Microstructure and mechanical property of graphene oxide/AlSi10Mg composites fabricated by laser additive manufacturing. Mater Charact.

[150] Hu Z., Chen F., Lin D., Nian Q., Parandoush P., Zhu X. (2017). Laser additive manufacturing bulk graphene-copper nanocomposites. Nanotechnology.

[151] Shuai C., Wang B., Yang Y., Peng S., Gao C. (2019). 3D honeycomb nanostructure-encapsulated magnesium alloys with superior corrosion resistance and mechanical properties. Compos. B Eng..

[152] Hu Z., Chen F., Xu J., Nian Q., Lin D., Chen C. (2018). 3D printing graphene-aluminum nanocomposites. J. Alloys Compd..

[153] Hu Z., Tong G., Lin D., Chen C., Guo H., Xu J. (2016). Graphene-reinforced metal matrix nanocomposites – a review. Mater. Sci. Technol..

[154] Fadavi Boostani A., Yazdani S., Taherzadeh Mousavian R., Tahamtan S., Azari Khosroshahi R., Wei D. (2015). Strengthening mechanisms of graphene sheets in aluminium matrix nanocomposites. Mater. Des..

[155] Ashwath P., Xavior M.A. (2014). The effect of ball milling & reinforcement percentage on sintered samples of aluminium alloy metal matrix composites. Procedia Eng..

[156] Lu G., Shi X., Liu X., Zhou H., Chen Y., Yang Z. (2019). Tribological performance of functionally gradient structure of graphene nanoplatelets reinforced Ni3Al metal matrix composites prepared by laser melting deposition. Wear.

[157] Wen S., Chen K., Li W., Zhou Y., Wei Q., Shi Y. (2019). Selective laser melting of reduced graphene oxide/S136 metal matrix composites with tailored microstructures and mechanical properties. Mater. Des..

[158] Wang Y., Shi J., Lu S., Wang Y. (2017). Selective laser melting of graphene-reinforced Inconel 718 superalloy: evaluation of microstructure and tensile performance. J. Manuf. Sci. Eng..

[159] Dadkhah M., Saboori A., Fino P. (2019). An overview of the recent developments in metal matrix nanocomposites reinforced by graphene. Materials.

[160] Goenka S., Sant V., Sant S. (2014). Graphene-based nanomaterials for drug delivery and tissue engineering. J. Contr. Release.

[161] Shin S.R., Zihlmann C., Akbari M., Assawes P., Cheung L., Zhang K. (2016). Reduced graphene oxide‐GelMA hybrid hydrogels as scaffolds for cardiac tissue engineering. Small.

[162] Hu S., Fang R., Chen Y., Liao B., Chen I., Chen S. (2014). Photoresponsive protein–graphene–protein hybrid capsules with dual targeted heat‐triggered drug delivery approach for enhanced tumor therapy. Adv. Funct. Mater..

[163] Chen L., Song L., Zhang Y., Wang P., Xiao Z., Guo Y. (2016). Nitrogen and sulfur codoped reduced graphene oxide as a general platform for rapid and sensitive fluorescent detection of biological species. ACS Appl. Mater. Interfaces.

[164] Wu J., Wang Y., Yang X., Liu Y., Yang J., Yang R. (2012). Graphene oxide used as a carrier for adriamycin can reverse drug resistance in breast cancer cells. Nanotechnology.

[165] Zhao H., Ding R., Zhao X., Li Y., Qu L., Pei H. (2017). Graphene-based nanomaterials for drug and/or gene delivery, bioimaging, and tissue engineering. Drug Discov. Today.

[166] Nie W., Peng C., Zhou X., Chen L., Wang W., Zhang Y. (2017). Three-dimensional porous scaffold by self-assembly of reduced graphene oxide and nano-hydroxyapatite composites for bone tissue engineering. Carbon N Y.

[167] Dubey N., Bentini R., Islam I., Cao T., Castro Neto A.H., Rosa V. (2015). Graphene: a versatile carbon-based material for bone tissue engineering. Stem Cells Int.

[168] Dinescu S., Ionita M., Pandele A.M., Galateanu B., Iovu H., Ardelean A. (2014). In vitro cytocompatibility evaluation of chitosan/graphene oxide 3D scaffold composites designed for bone tissue engineering. Bio Med. Mater. Eng..

[169] Unnithan A.R., Park C.H., Kim C.S. (2016). Nanoengineered bioactive 3D composite scaffold: a unique combination of graphene oxide and nanotopography for tissue engineering applications. Compos. B Eng..

[170] Yu P., Bao R.-Y., Shi X.-J., Yang W., Yang M.-B. (2017). Self-assembled high-strength hydroxyapatite/graphene oxide/chitosan composite hydrogel for bone tissue engineering. Carbohydr. Polym..

[171] Wang W., Caetano G., Ambler W., Blaker J., Frade M., Mandal P. (2016). Enhancing the hydrophilicity and cell attachment of 3D printed PCL/graphene scaffolds for bone tissue engineering. Materials.

[172] Li J., Liu X., Crook J.M., Wallace G.G. (2019). 3D graphene-containing structures for tissue engineering. Mater. Today Chem..

[173] Jakus A.E., Shah R.N. (2017). Multi and mixed 3D-printing of graphene-hydroxyapatite hybrid materials for complex tissue engineering. J. Biomed. Mater. Res..

[174] Nosrati H., Sarraf Mamoory R., Svend Le D.Q., Bünger C.E. (2020). Fabrication of gelatin/hydroxyapatite/3D-graphene scaffolds by a hydrogel 3D-printing method. Mater. Chem. Phys..

[175] Jakus A.E., Secor E.B., Rutz A.L., Jordan S.W., Hersam M.C., Shah R.N. (2015). Three-dimensional printing of high-content graphene scaffolds for electronic and biomedical applications. ACS Nano.

[176] Shuai C., Liu T., Gao C., Feng P., Xiao T., Yu K. (2016). Mechanical and structural characterization of diopside scaffolds reinforced with graphene. J. Alloys Compd..

[177] Khan Z.U., Kausar A., Ullah H., Badshah A., Khan W.U. (2016). A review of graphene oxide, graphene buckypaper, and polymer/graphene composites: properties and fabrication techniques. J. Plast. Film Sheeting.

[178] Li Y., Wang S., Wang Q., Xing M. (2018). A comparison study on mechanical properties of polymer composites reinforced by carbon nanotubes and graphene sheet. Compos. B Eng..

[179] Su Y., Zhitomirsky I. (2013). Electrophoretic deposition of graphene, carbon nanotubes and composite films using methyl violet dye as a dispersing agent. Colloids Surf. A Physicochem. Eng. Asp..

[180] Kim J.H., Chang W.S., Kim D., Yang J.R., Han J.T., Lee G. (2015). 3D printing of reduced graphene oxide nanowires. Adv. Mater..

[181] Mohan V.B., Lau K., Hui D., Bhattacharyya D. (2018). Graphene-based materials and their composites: a review on production, applications and product limitations. Compos. B Eng..

[182] Idowu A., Boesl B., Agarwal A. (2018). 3D graphene foam-reinforced polymer composites – a review. Carbon N Y.

